# Correction: Development and analyses of stakeholder driven conceptual models to support the implementation of ecosystem-based fisheries management in the U.S. Caribbean

**DOI:** 10.1371/journal.pone.0320882

**Published:** 2025-03-19

**Authors:** Tarsila Seara, Stacey M. Williams, Kiara Acevedo, Graciela Garcia-Molliner, Orian Tzadik, Michelle Duval, Juan J. Cruz-Motta

The following information is missing from the Acknowledgements section: The authors would like to thank Vanessa Ramirez, Nicole Angeli, and Sennai Habtes for directly helping with workshop outreach and logistics. The success of the stakeholder workshops depended on the work of many collaborators (too many to list here) and we are equally grateful to all who provided their assistance and support. Finally, a special thanks to all workshop participants, especially the fishers, who took the time to engage and help us better understand the U.S. Caribbean ecosystem.
