# Correction: Development and analyses of stakeholder driven conceptual models to support the implementation of ecosystem-based fisheries management in the U.S. Caribbean

**DOI:** 10.1371/journal.pone.0334558

**Published:** 2025-10-14

**Authors:** Tarsila Seara, Stacey M. Williams, Kiara Acevedo, Graciela Garcia-Moliner, Orian Tzadik, Michelle Duval, Juan J. Cruz-Motta

The fourth author’s name is spelled incorrectly. The correct name is: Graciela Garcia-Moliner. The correct citation is: Seara T, Williams SM, Acevedo K, Garcia-Moliner G, Tzadik O, Duval M, et al. (2024) Development and analyses of stakeholder driven conceptual models to support the implementation of ecosystem-based fisheries management in the U.S. Caribbean. PLoS ONE 19(5): e0304101. https://doi.org/10.1371/journal.pone.0304101
