# Supplementary material for: Development and analyses of stakeholder driven conceptual models to support the implementation of ecosystem-based fisheries management in the U.S. Caribbean
Source: PLoS One. 2024 May 31;19(5):e0304101. doi: 10.1371/journal.pone.0304101 (PMC11142612; doi:10.1371/journal.pone.0304101)
Supplement: S1 File — (DOCX) [file pone.0304101.s002.docx]

# Supporting Information 1

## Conceptual Models for the U.S. Caribbean by Stakeholder Group


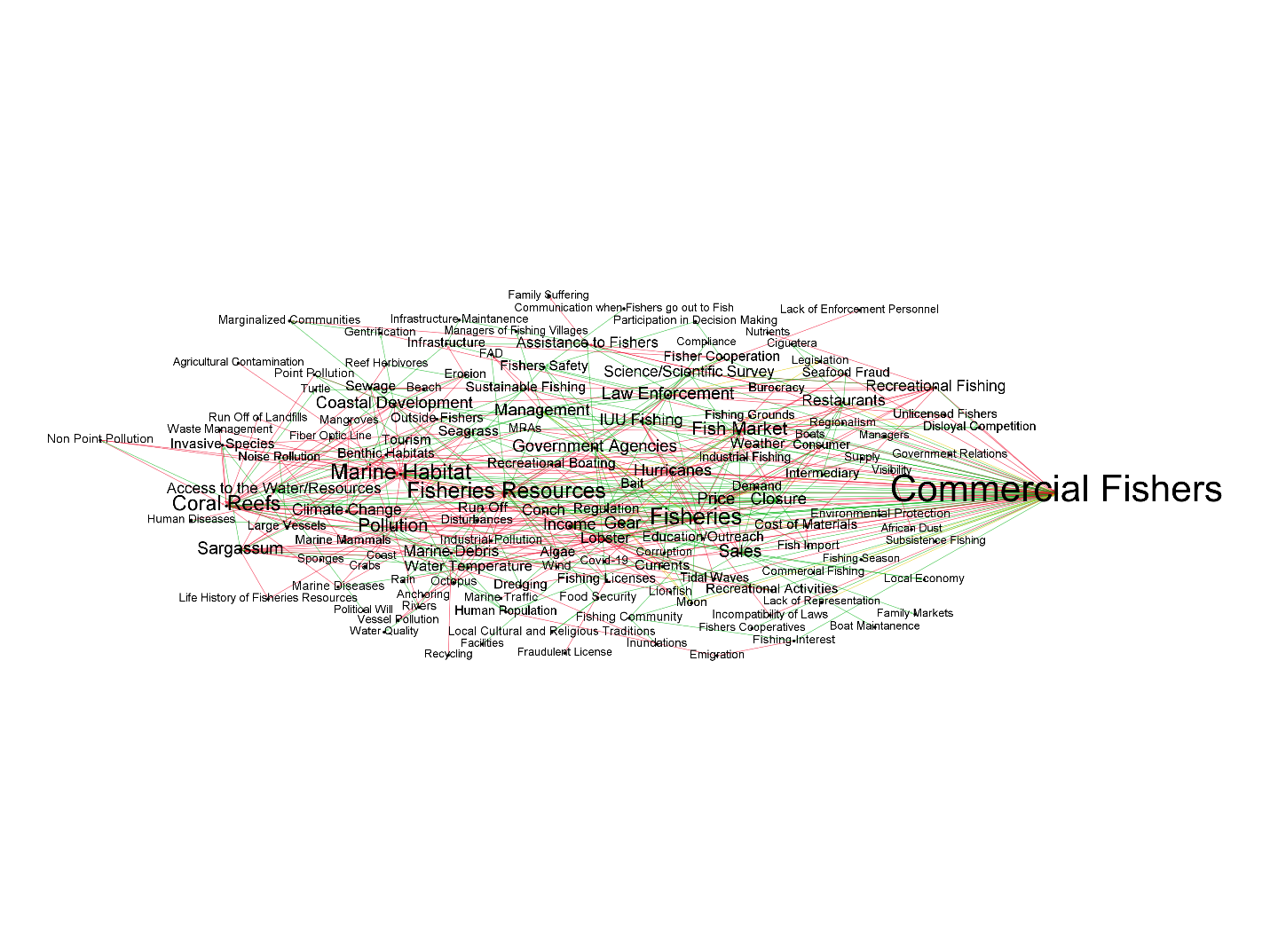


**S1 Fig 1. Fishers Conceptual Model.**


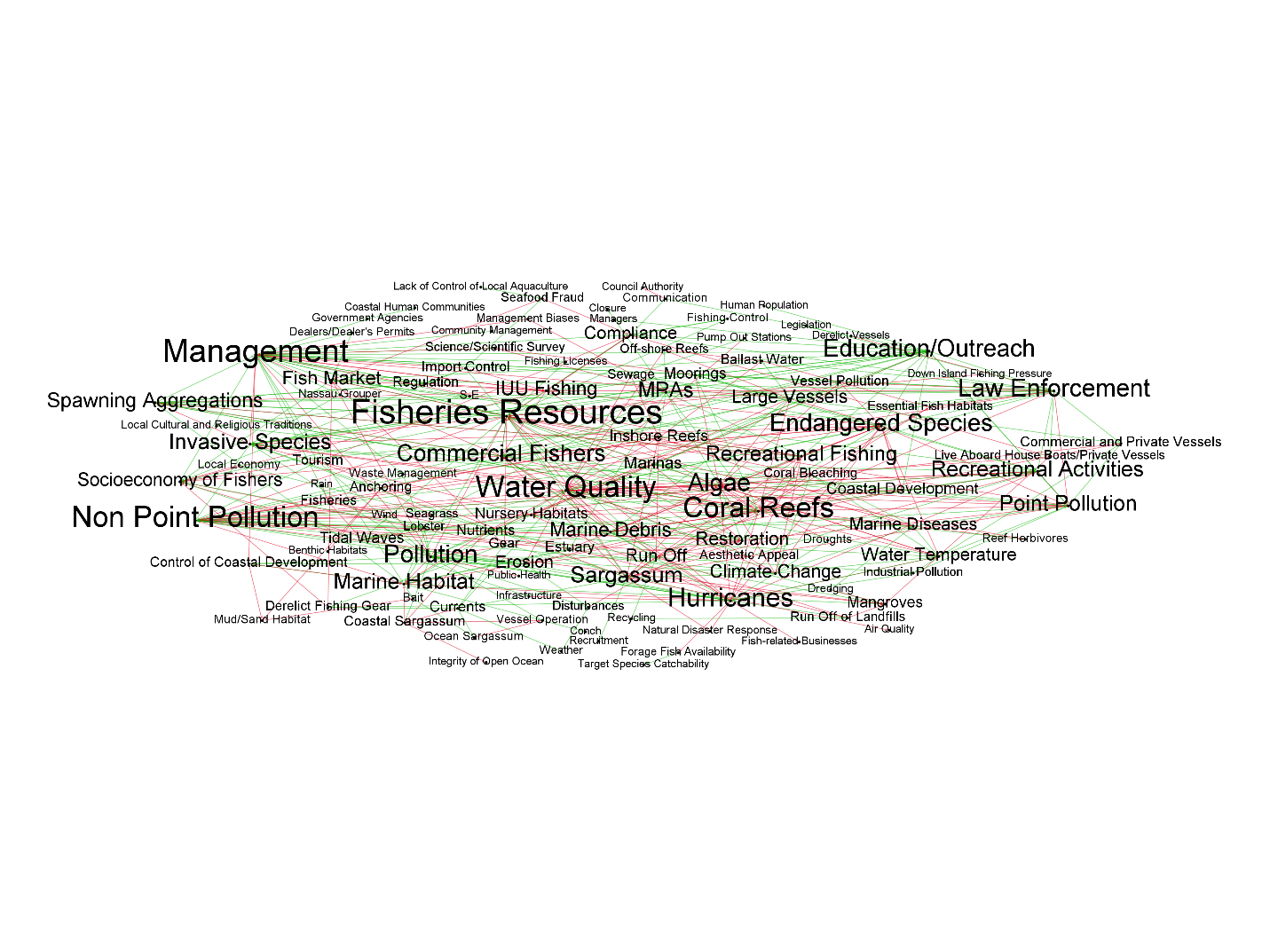


**S1 Fig 2. DAPs Conceptual Model.**


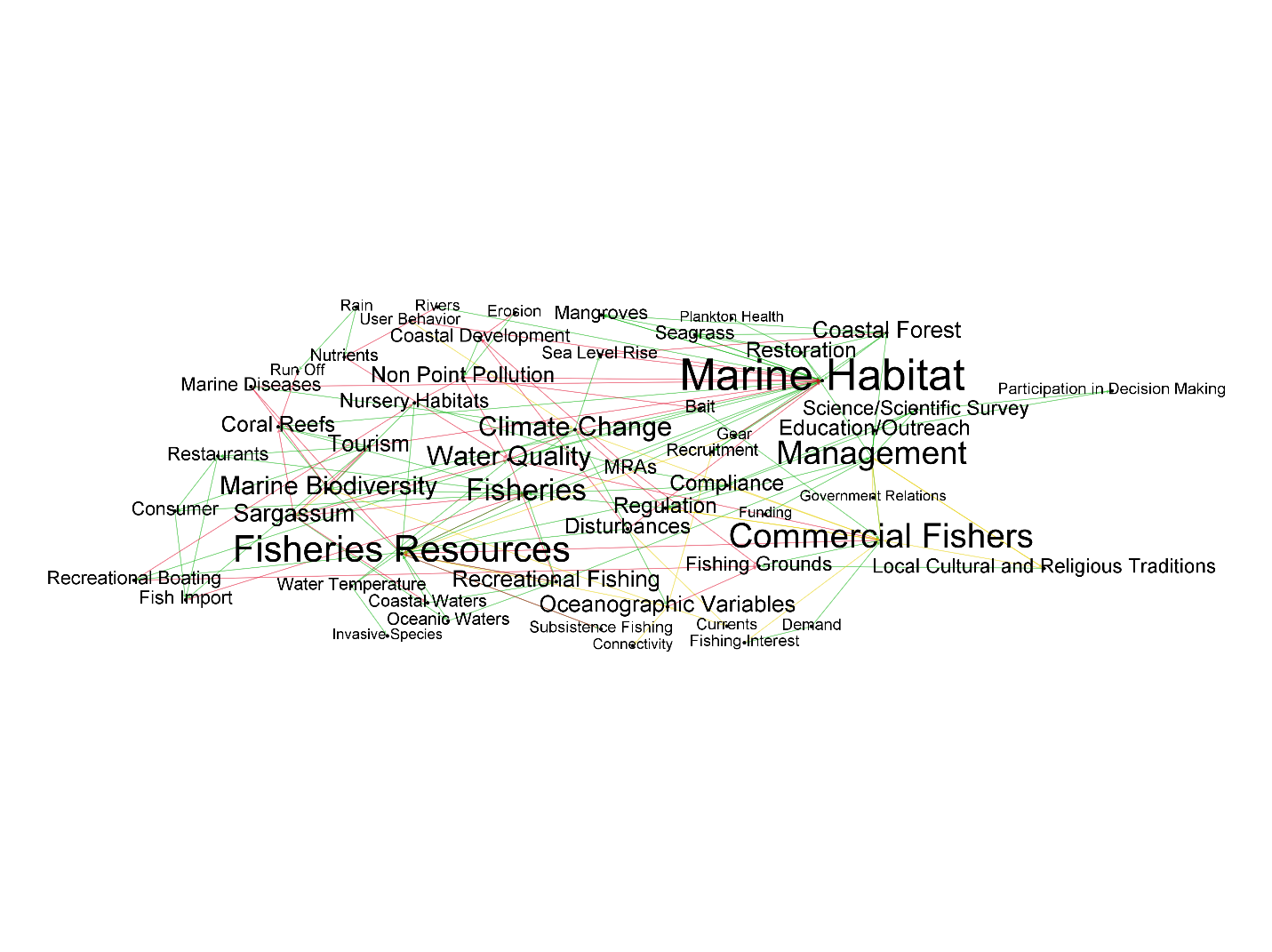


**S1 Fig 3. Managers Conceptual Model.**


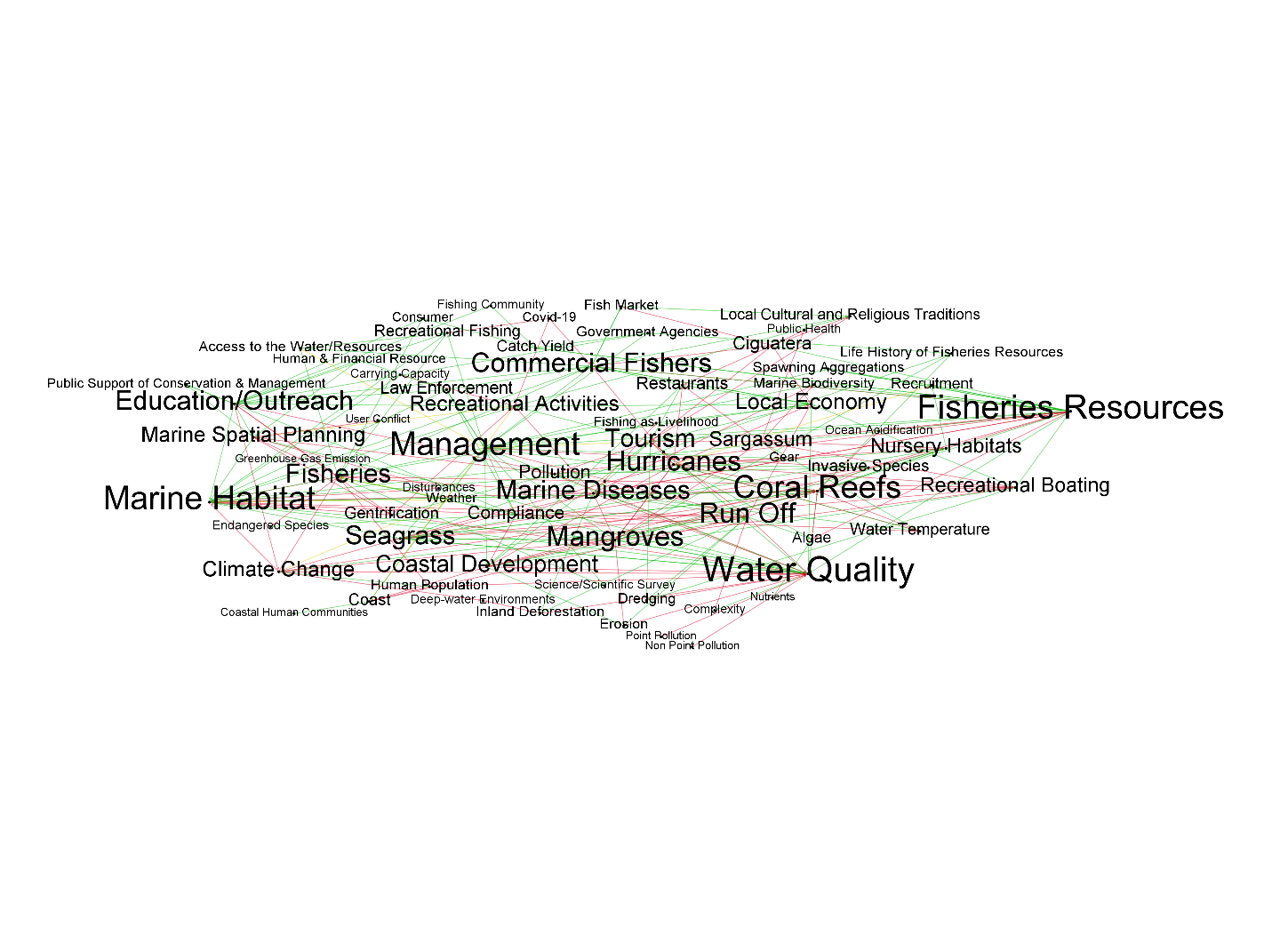


**S1 Fig 4. Academics Conceptual Model.**


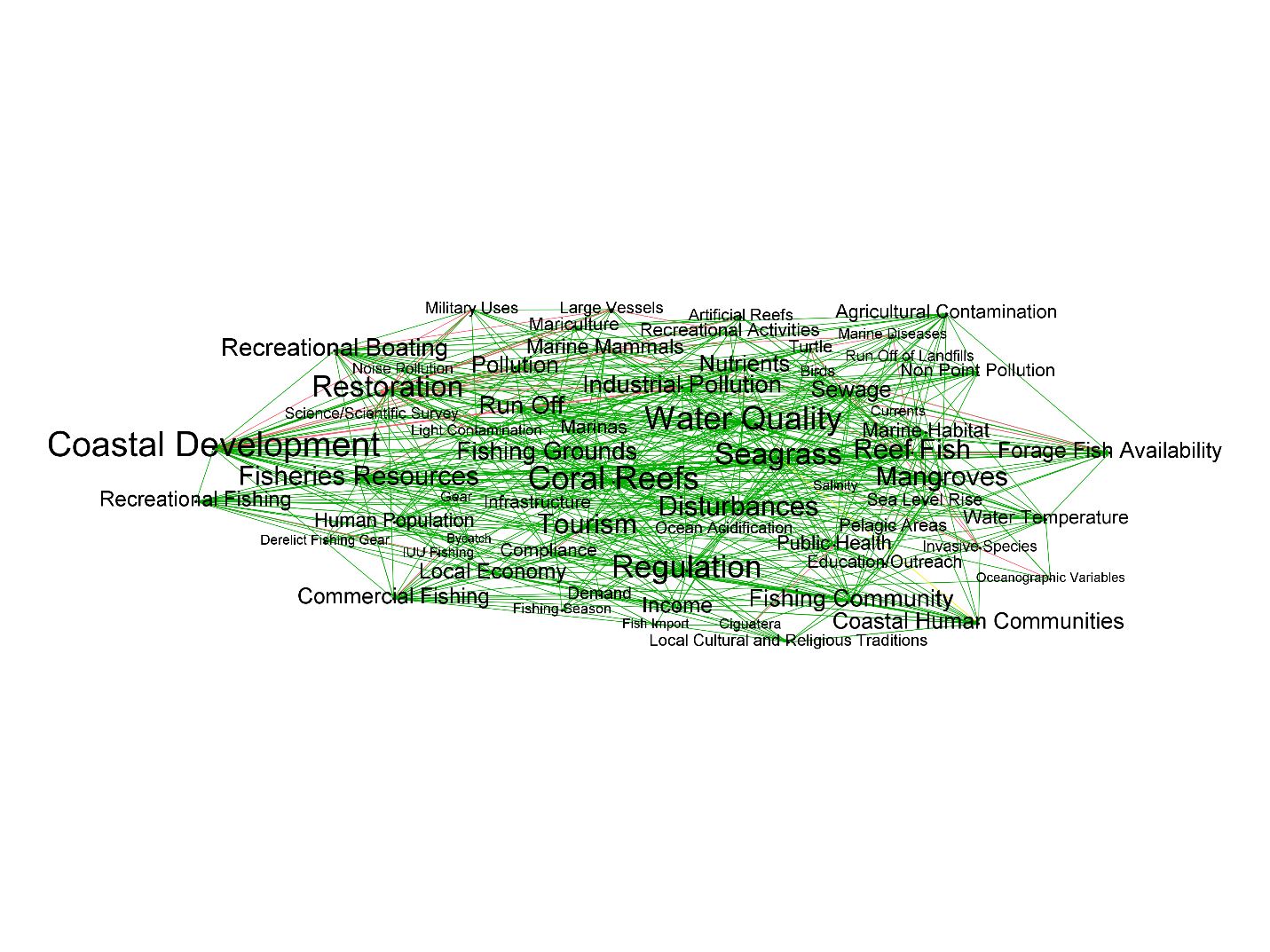


**S1 Fig 5. SSC Conceptual Model.**


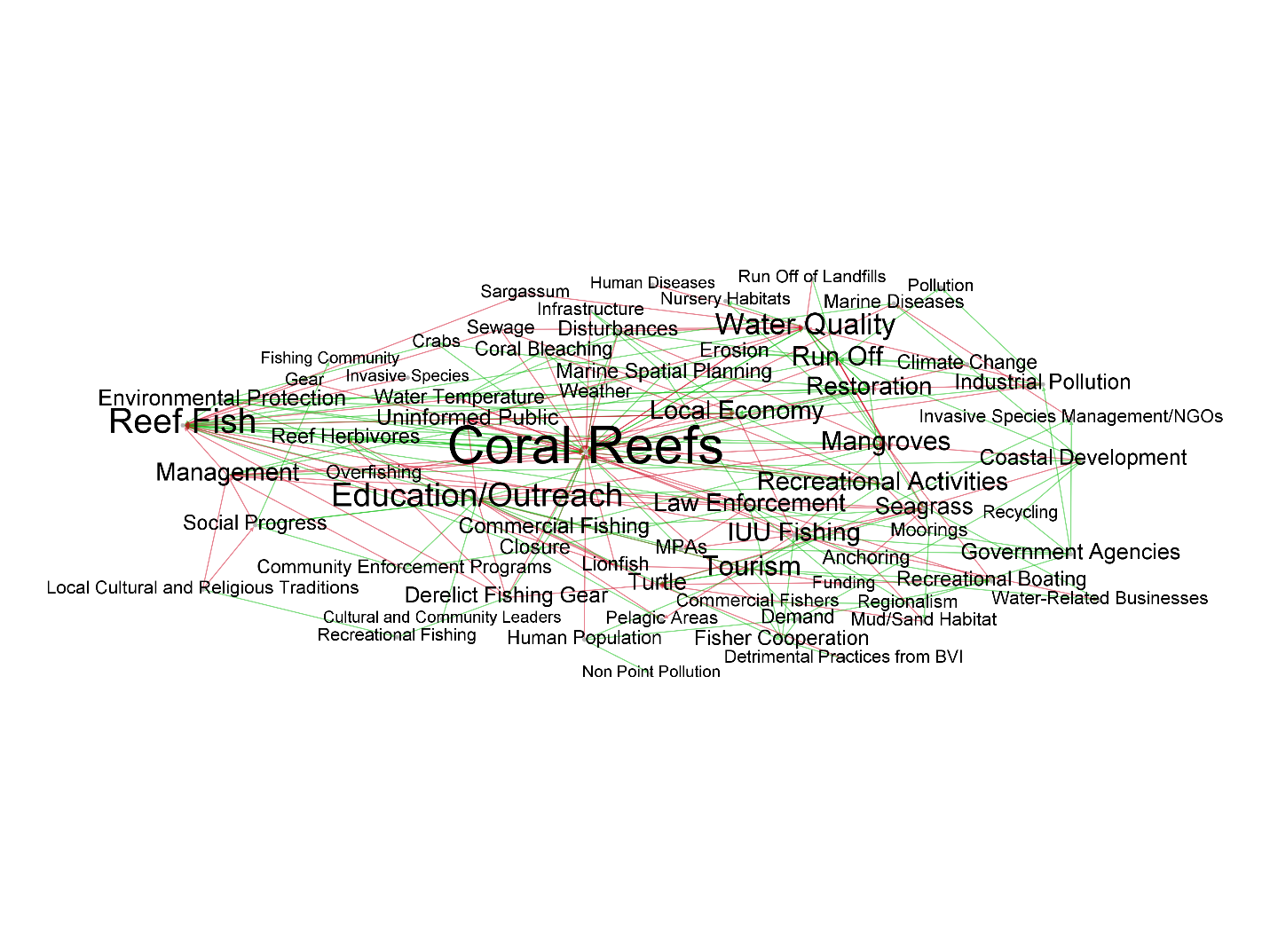


**S1 Fig 6. Businesses Conceptual Model.**


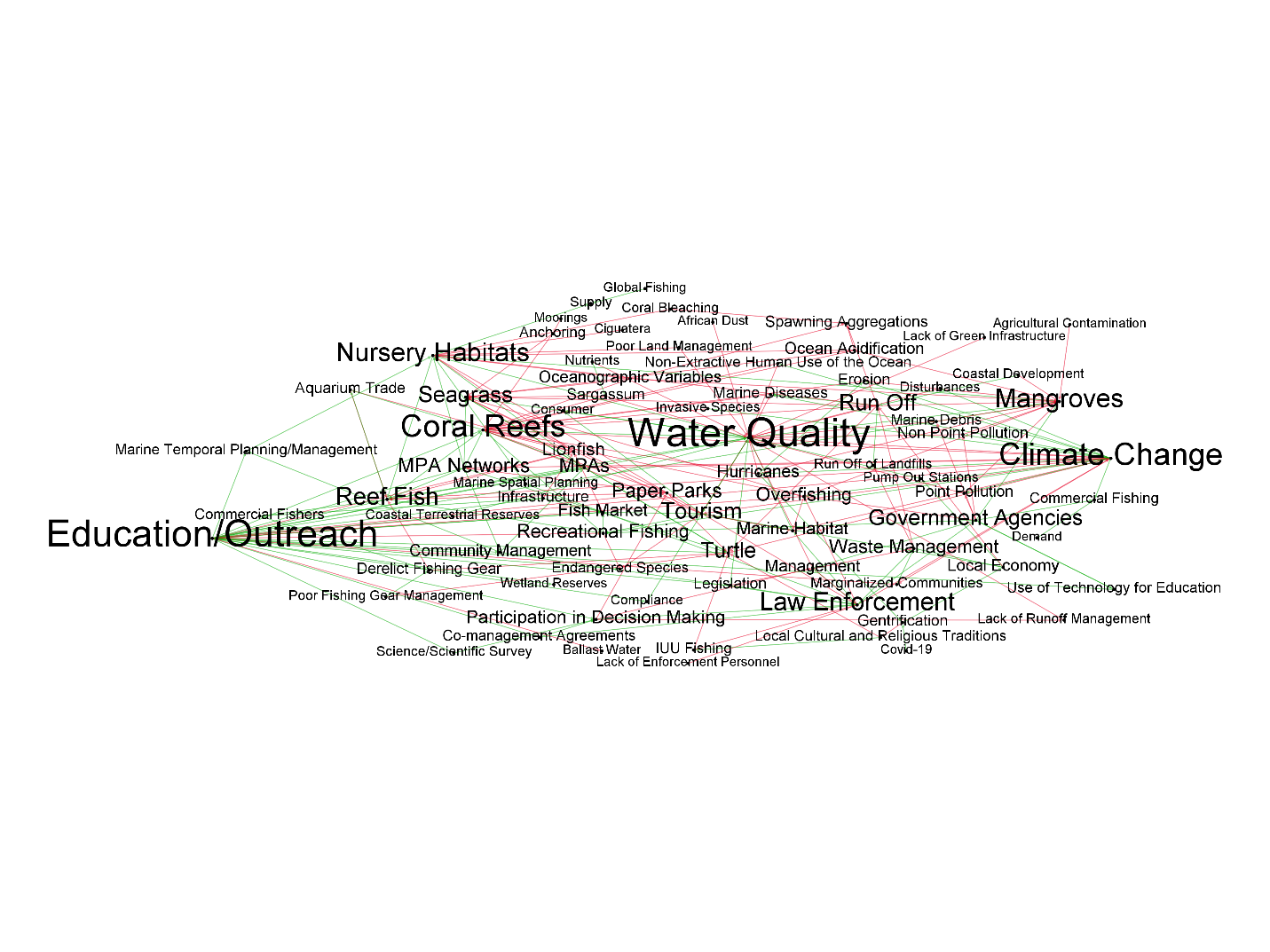


**S1 Fig 7. NGOs Conceptual Model.**

## Conceptual Models by Stakeholder Group by Island/Island Group

### Puerto Rico


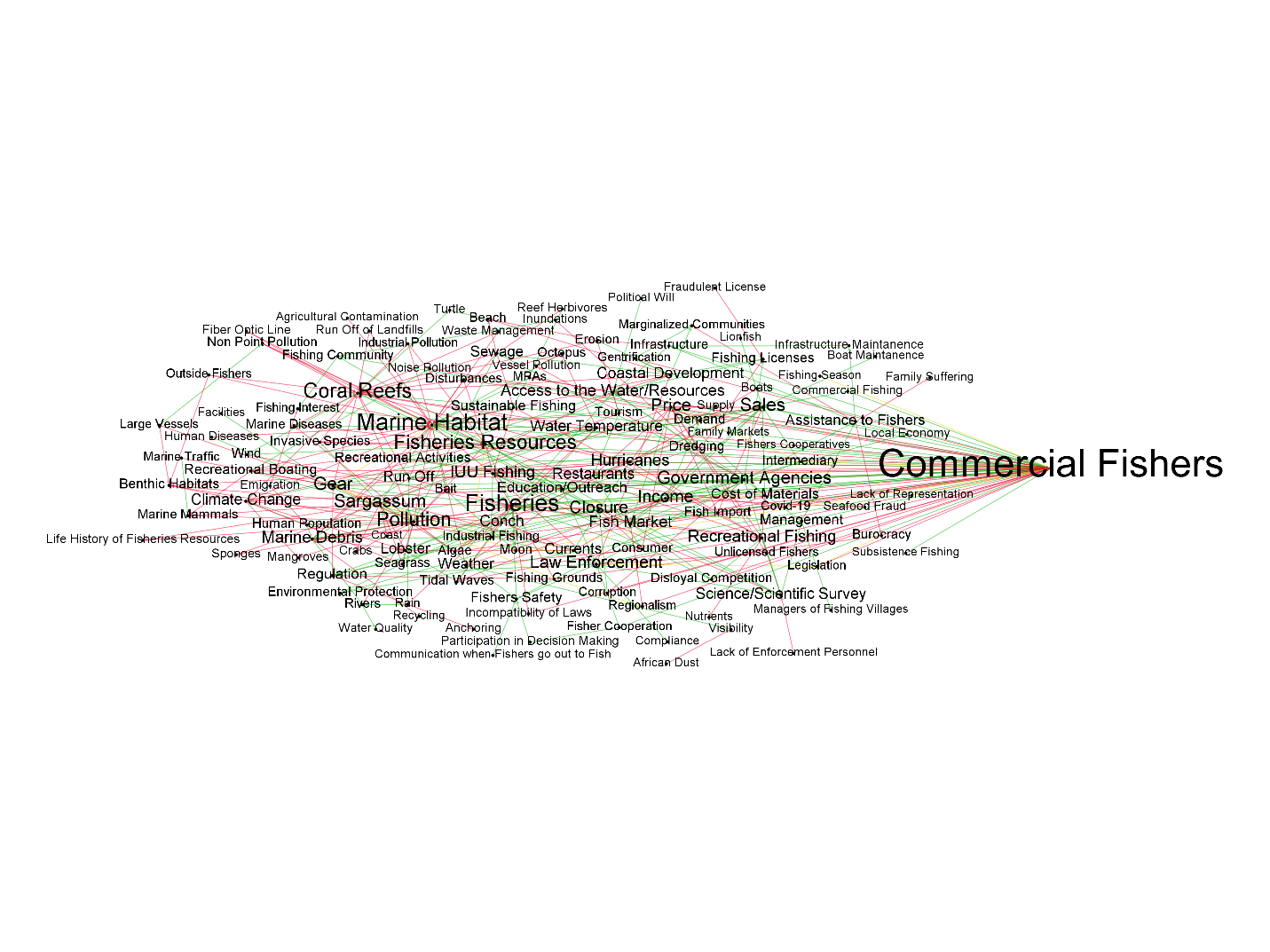


**S1 Fig 8. Puerto Rico Fishers Conceptual Model**


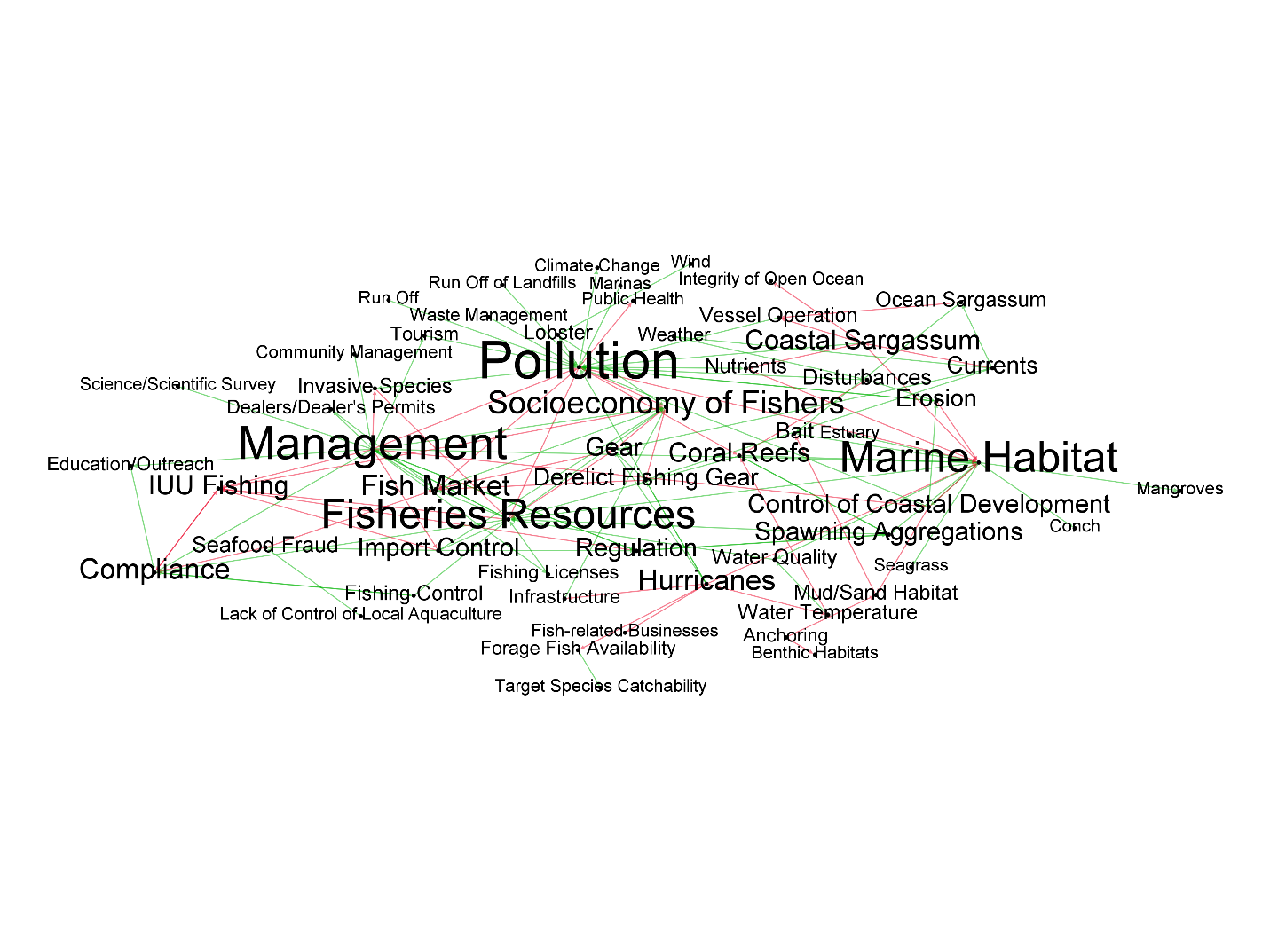


**S1 Fig 9. Puerto Rico DAP Conceptual Model**


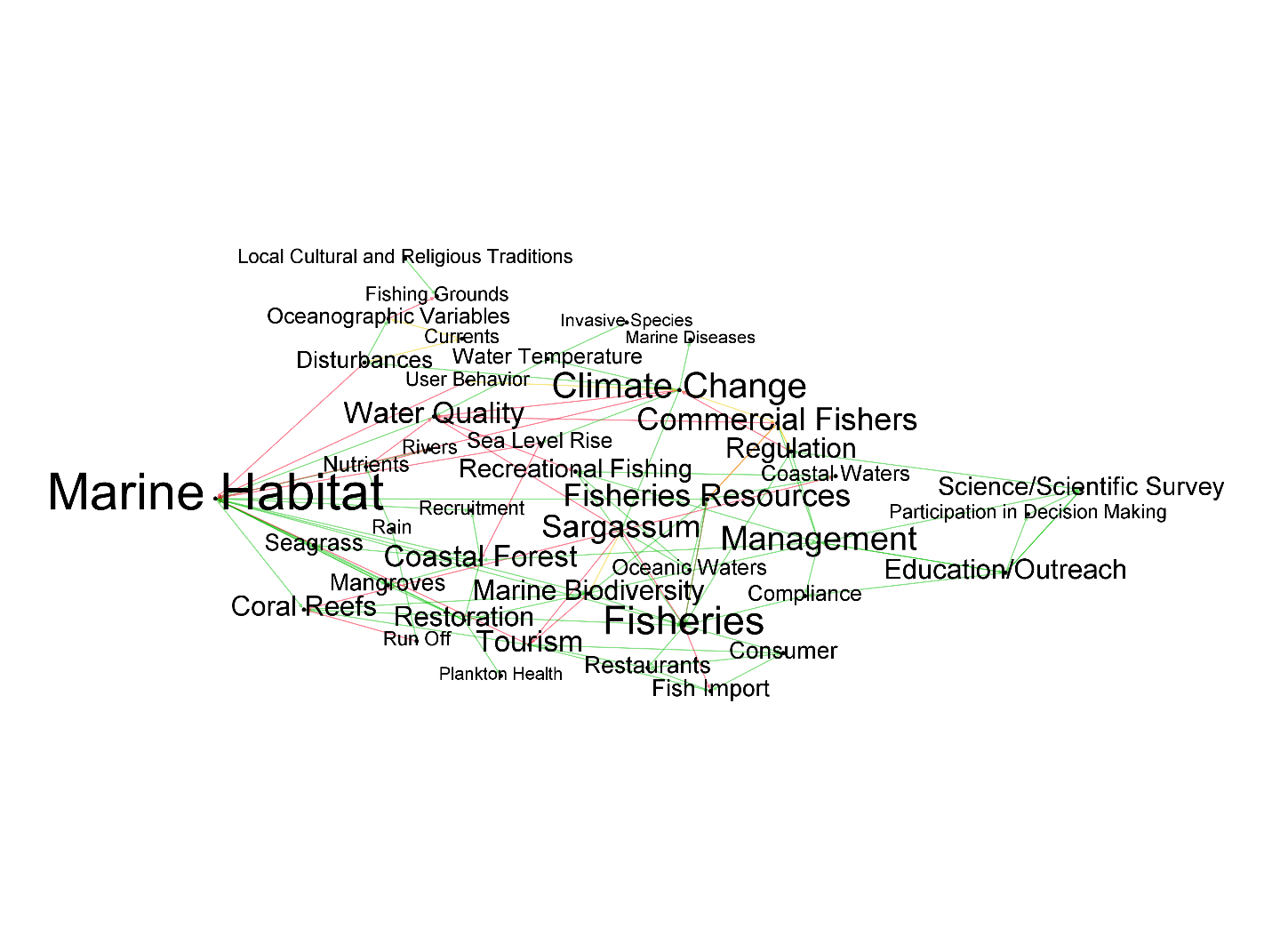


**S1 Fig 10. Puerto Rico Managers Conceptual Model.**


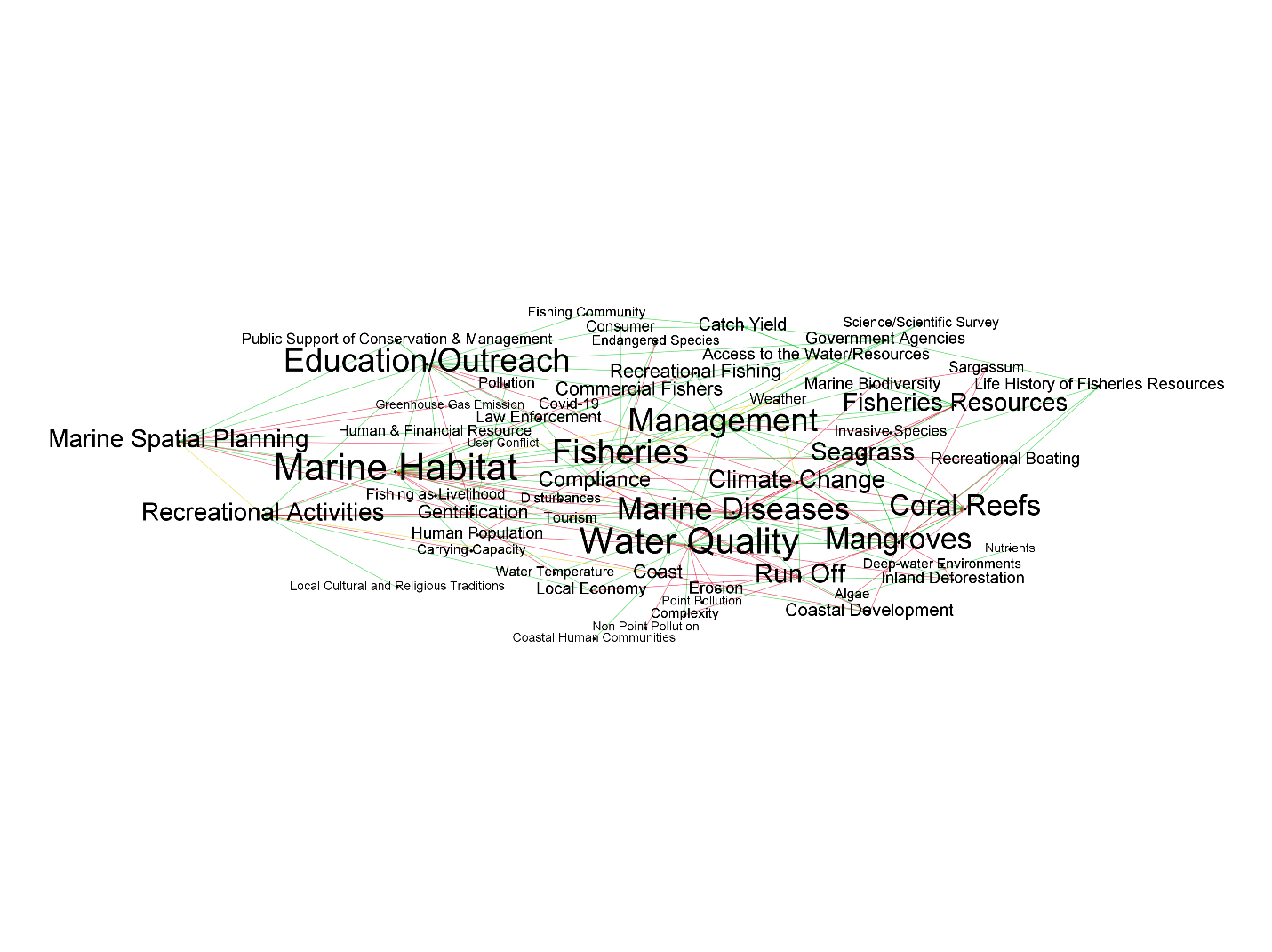


**S1 Fig 11. Puerto Rico Academics Conceptual Model.**


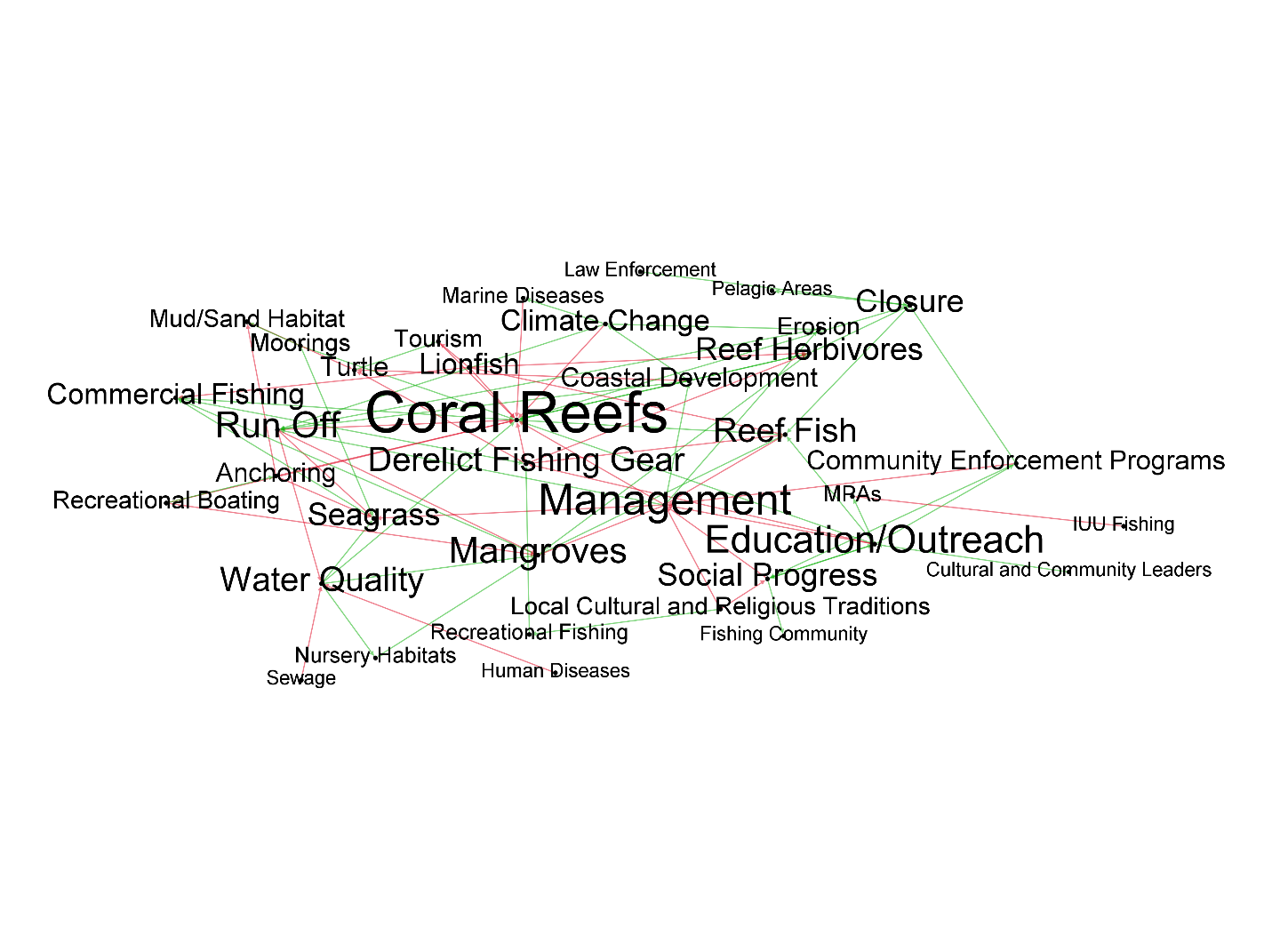


**S1 Fig 12. Puerto Rico Businesses Conceptual Model.**


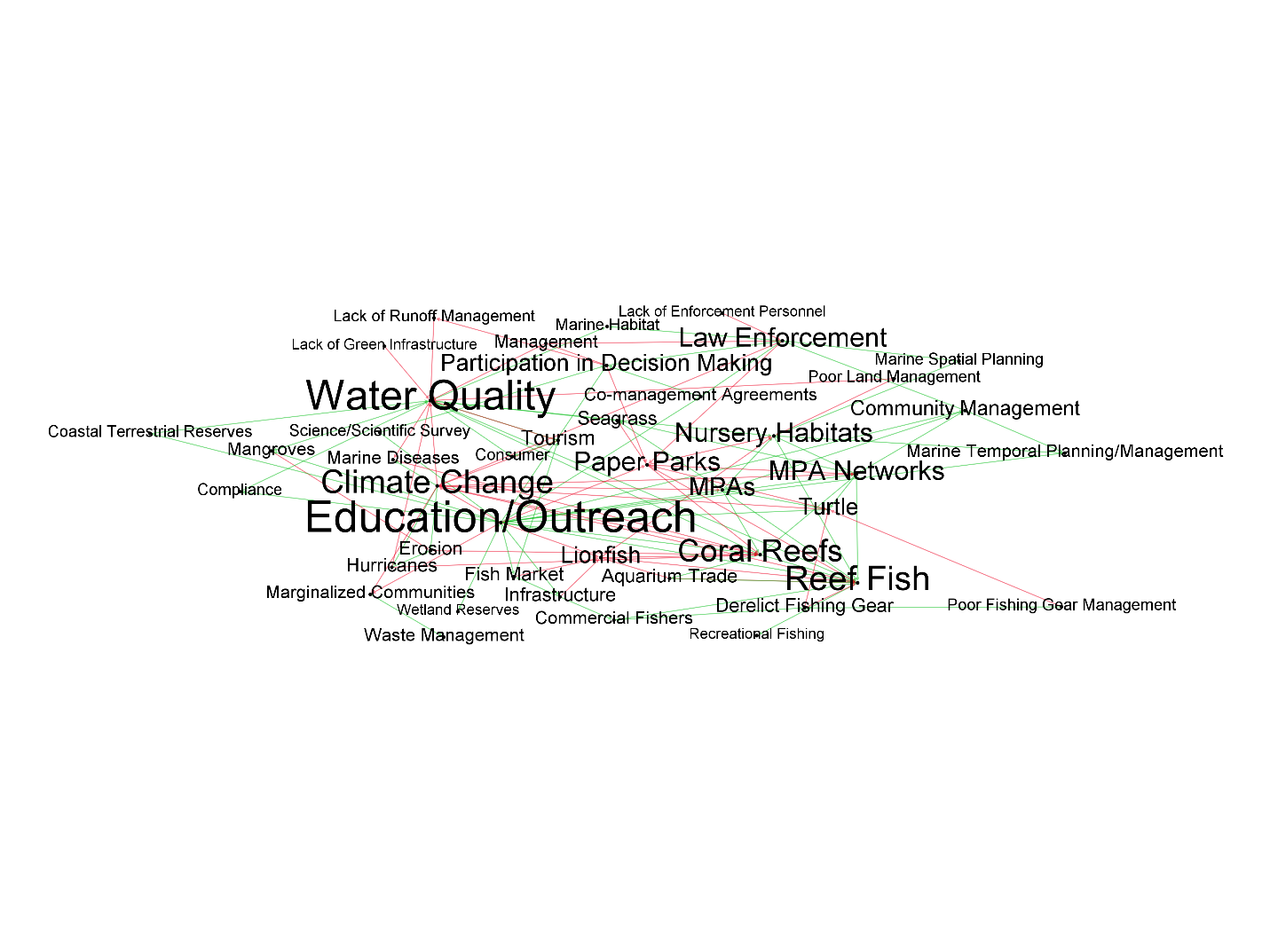


**S1 Fig 13. Puerto Rico NGOs Conceptual Model.**

### U.S. Virgin Islands


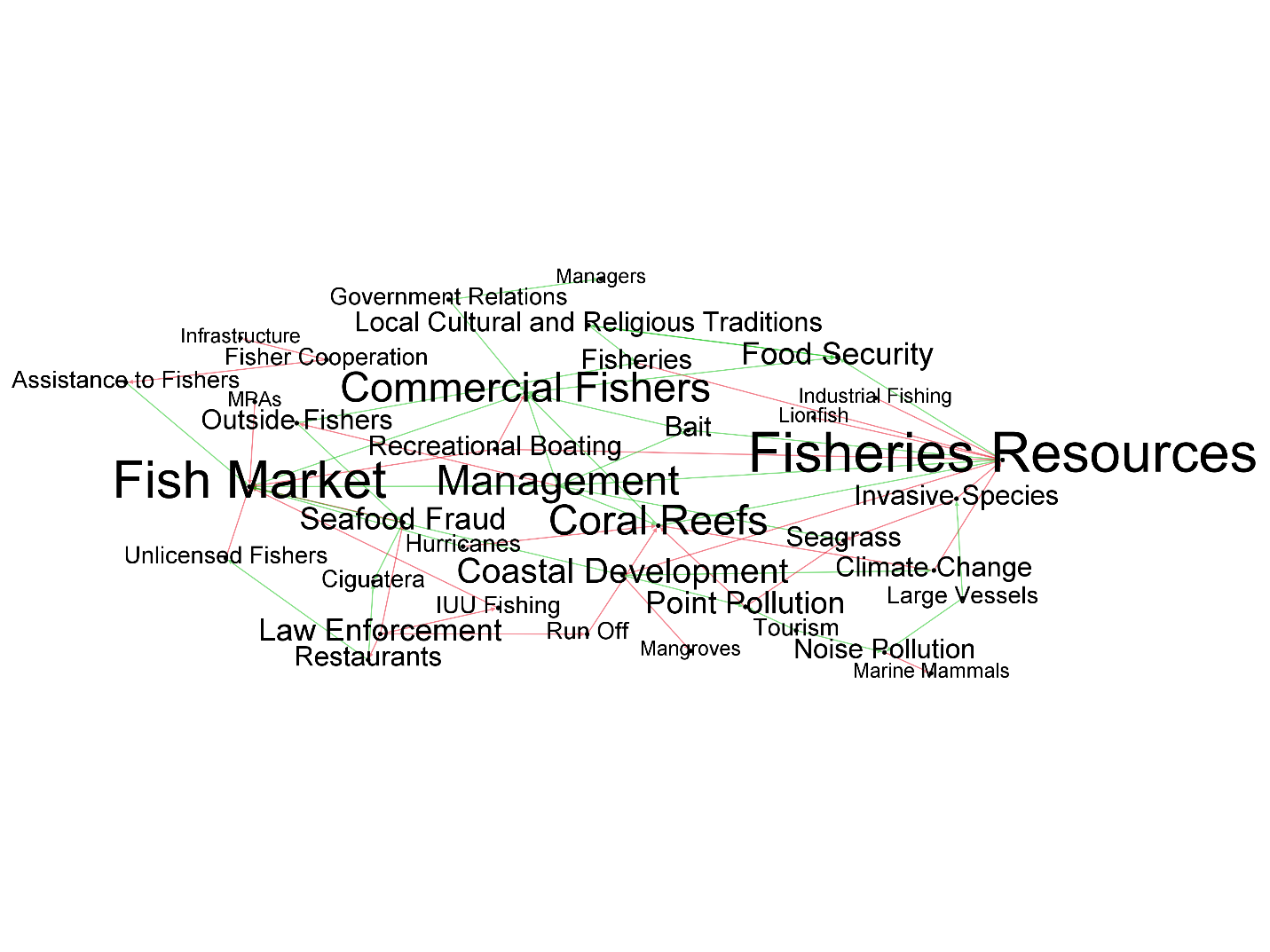


**S1 Fig 14. St. Thomas/St. John Fishers’ Conceptual Model.**


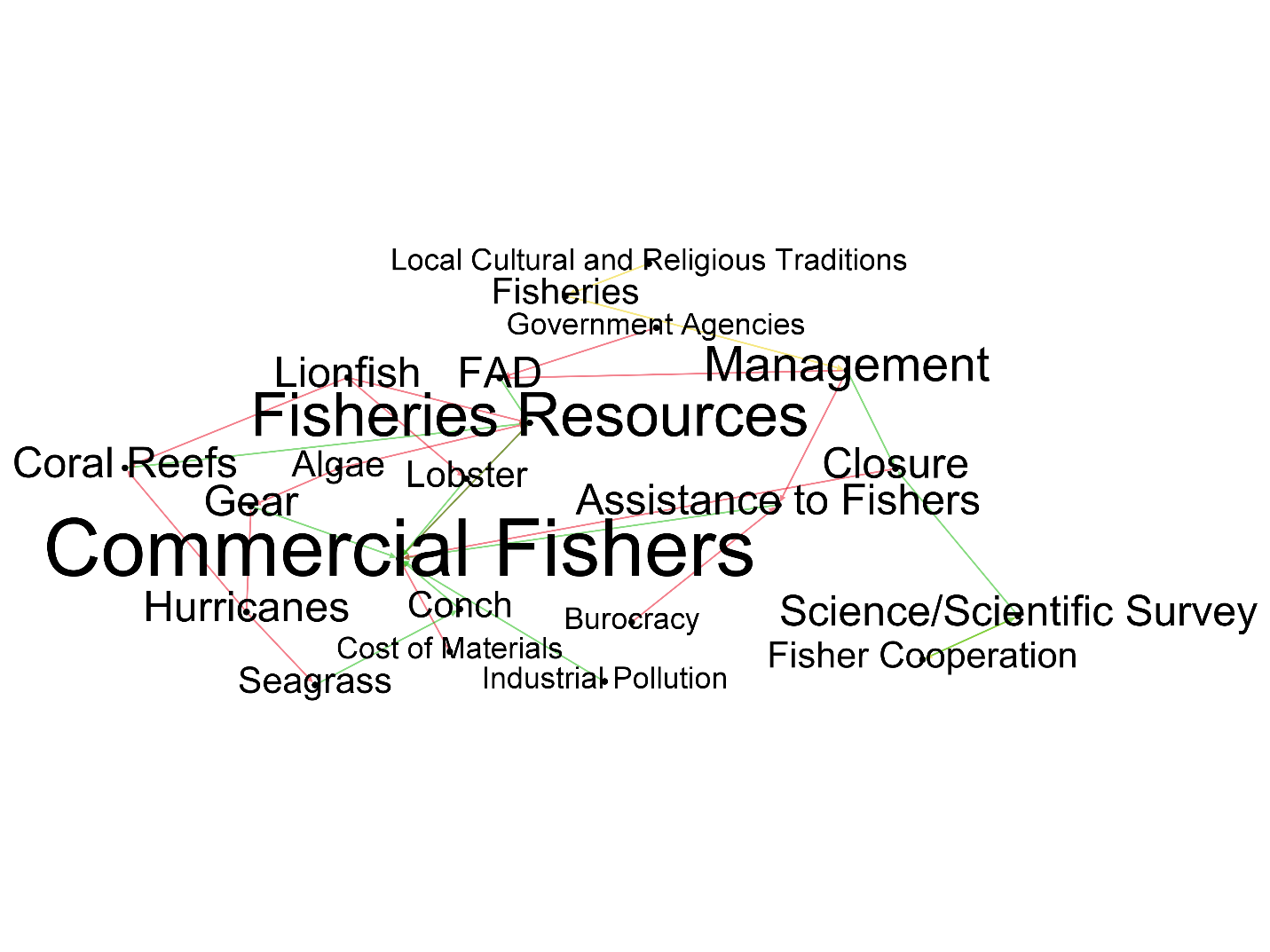


**S1 Fig 15. St. Croix Fishers’ Conceptual Model.**


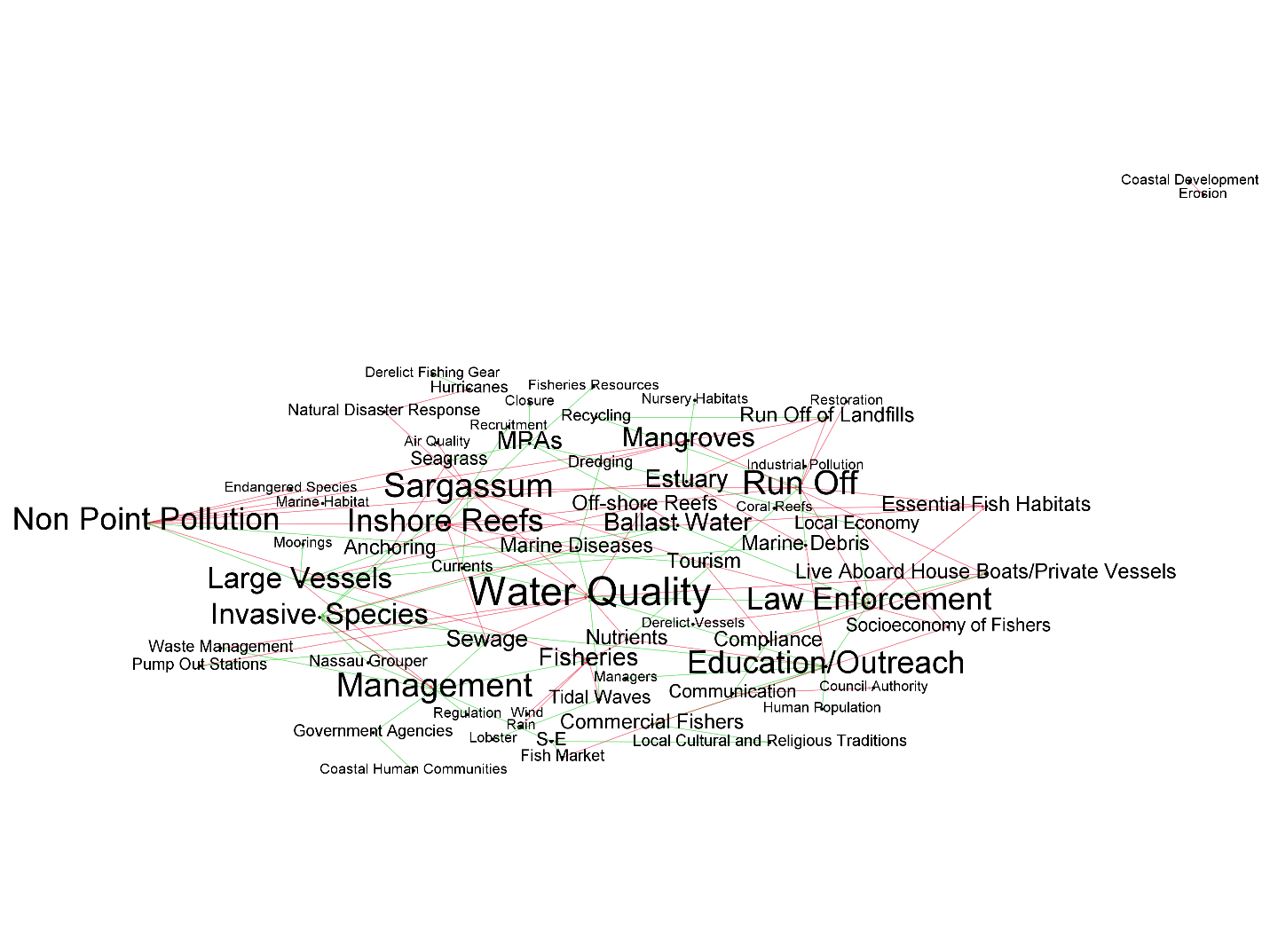


**S1 Fig 16. St. Thomas/St. John DAP Conceptual Model.**


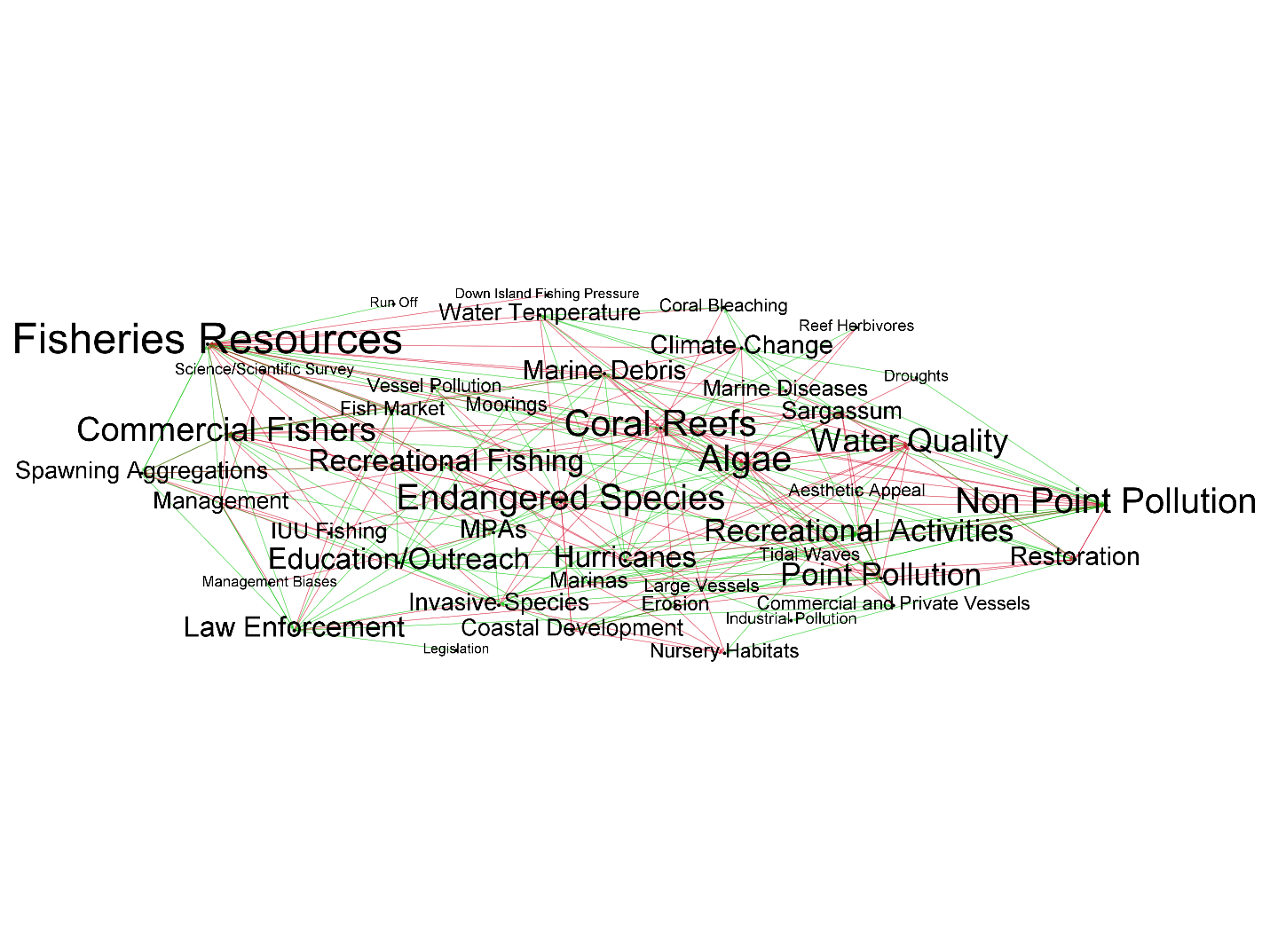


**S1 Fig 17. St. Croix DAP Conceptual Model.**


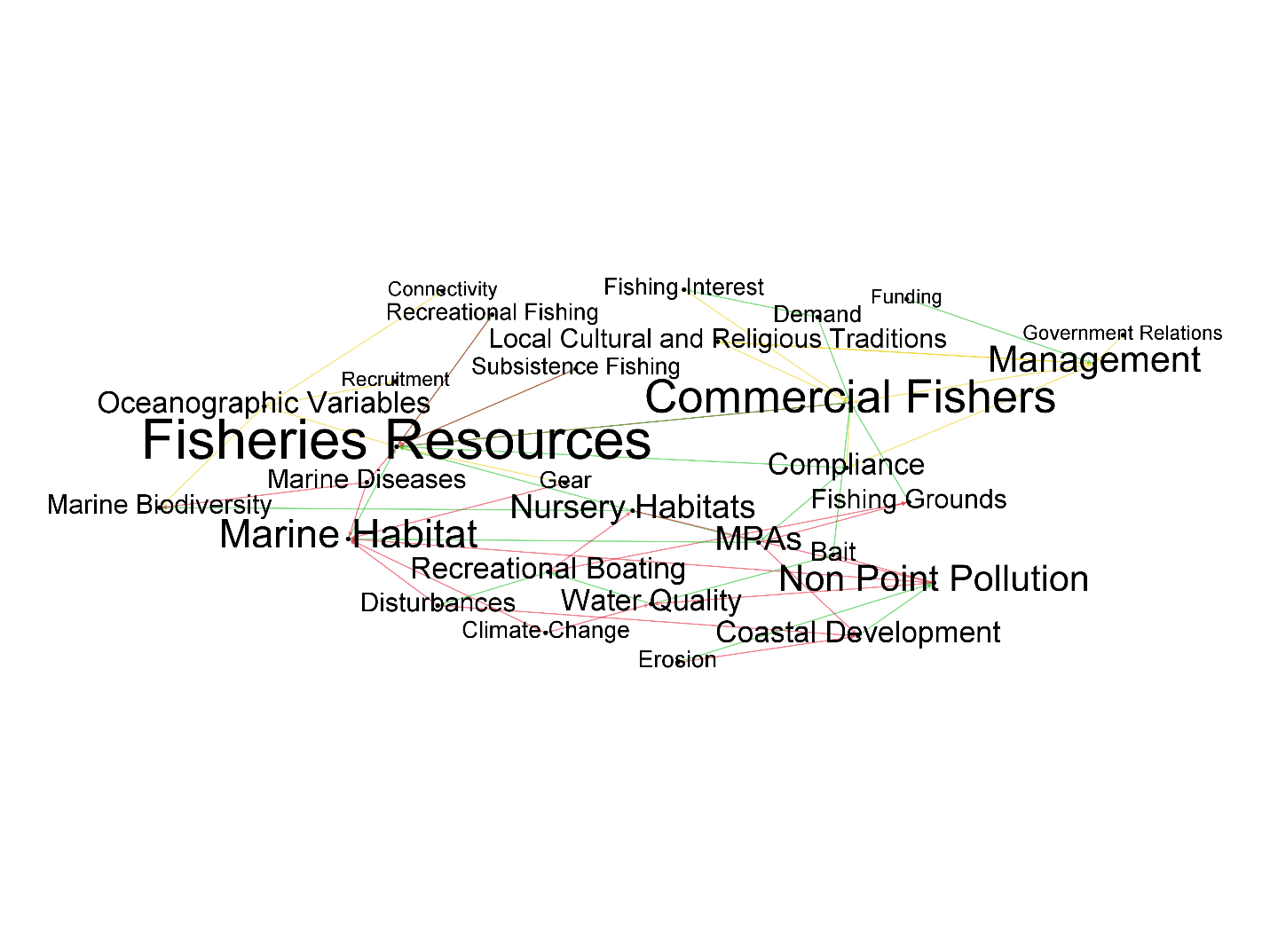


**S1 Fig 18. USVI Managers Conceptual Model.**


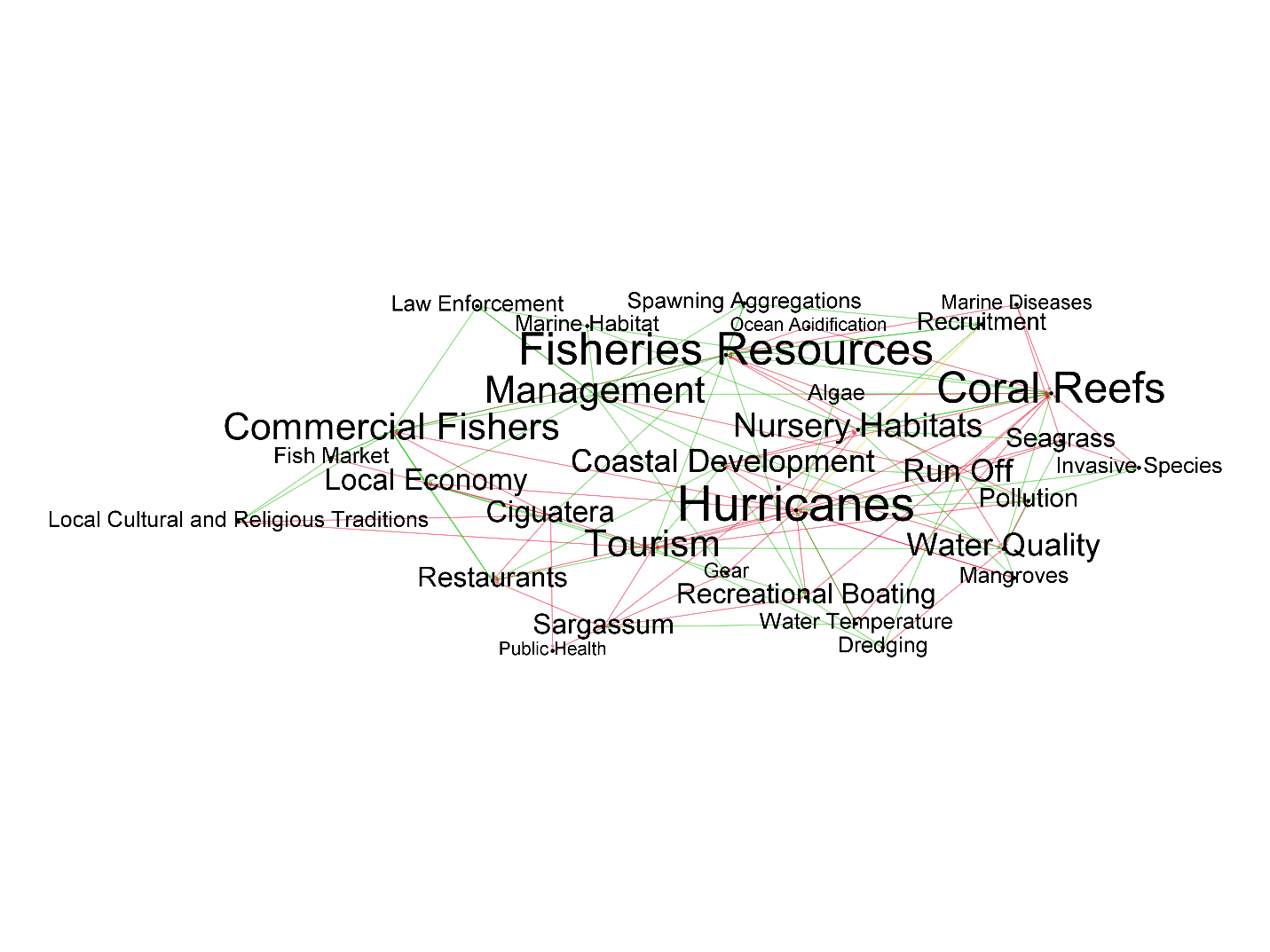


**S1 Fig 19. USVI Academics Conceptual Model.**


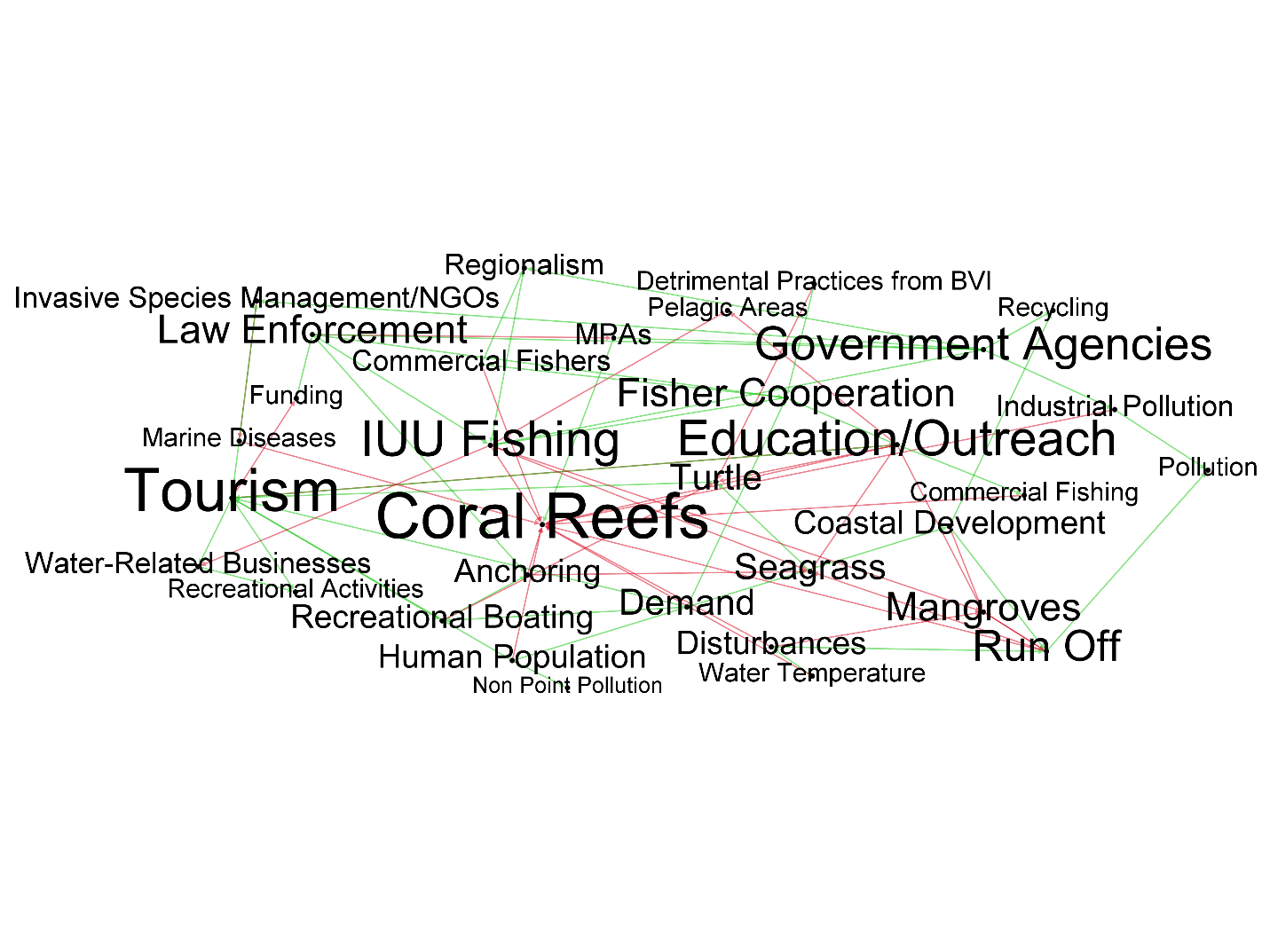


**S1 Fig 20. St. Thomas/St. John Businesses Conceptual Model.**


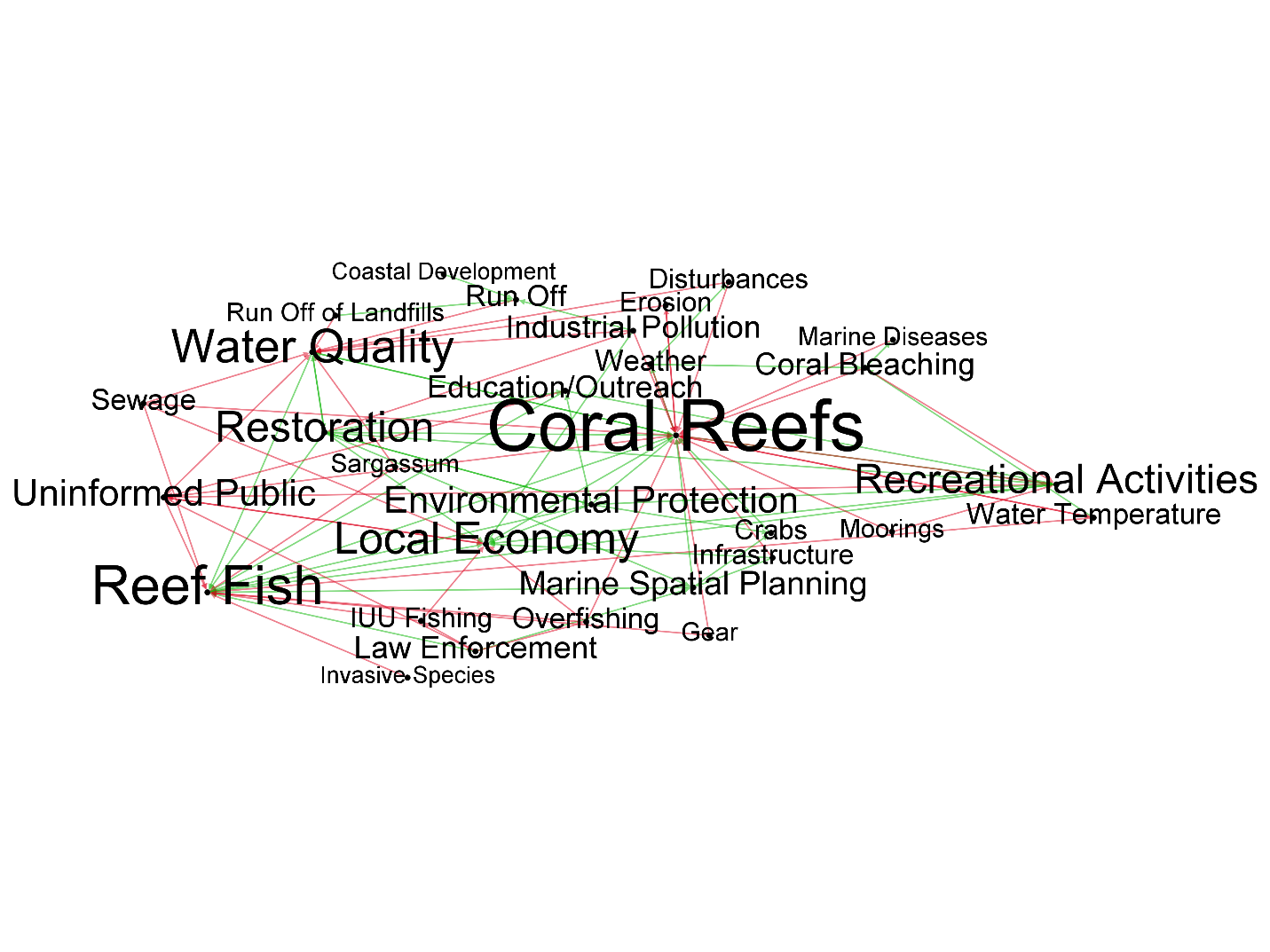


**S1 Fig 21. St. Croix Businesses Conceptual Model.**


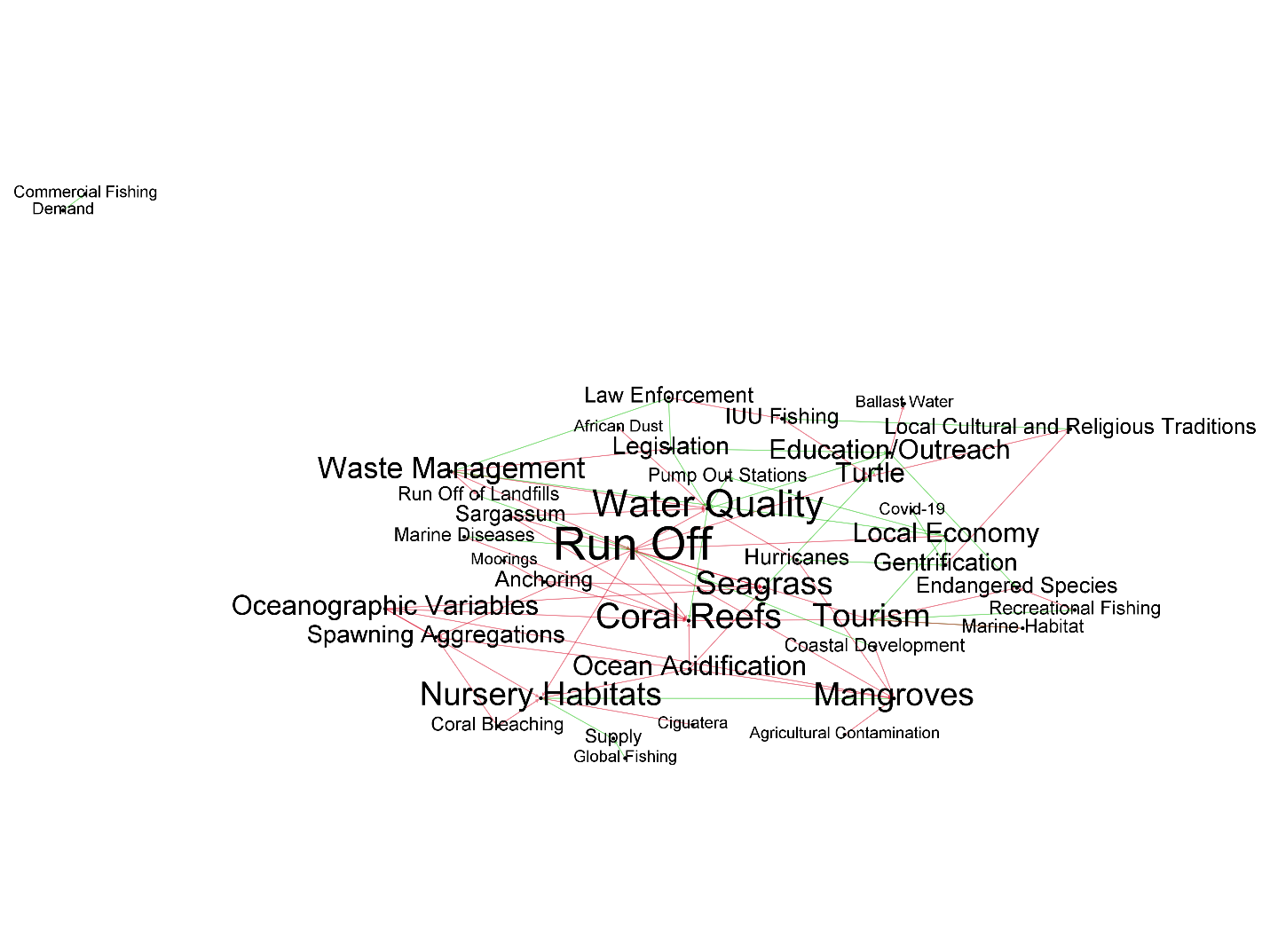


**S1 Fig 22. St. Thomas/St. John NGOs Conceptual Model.**


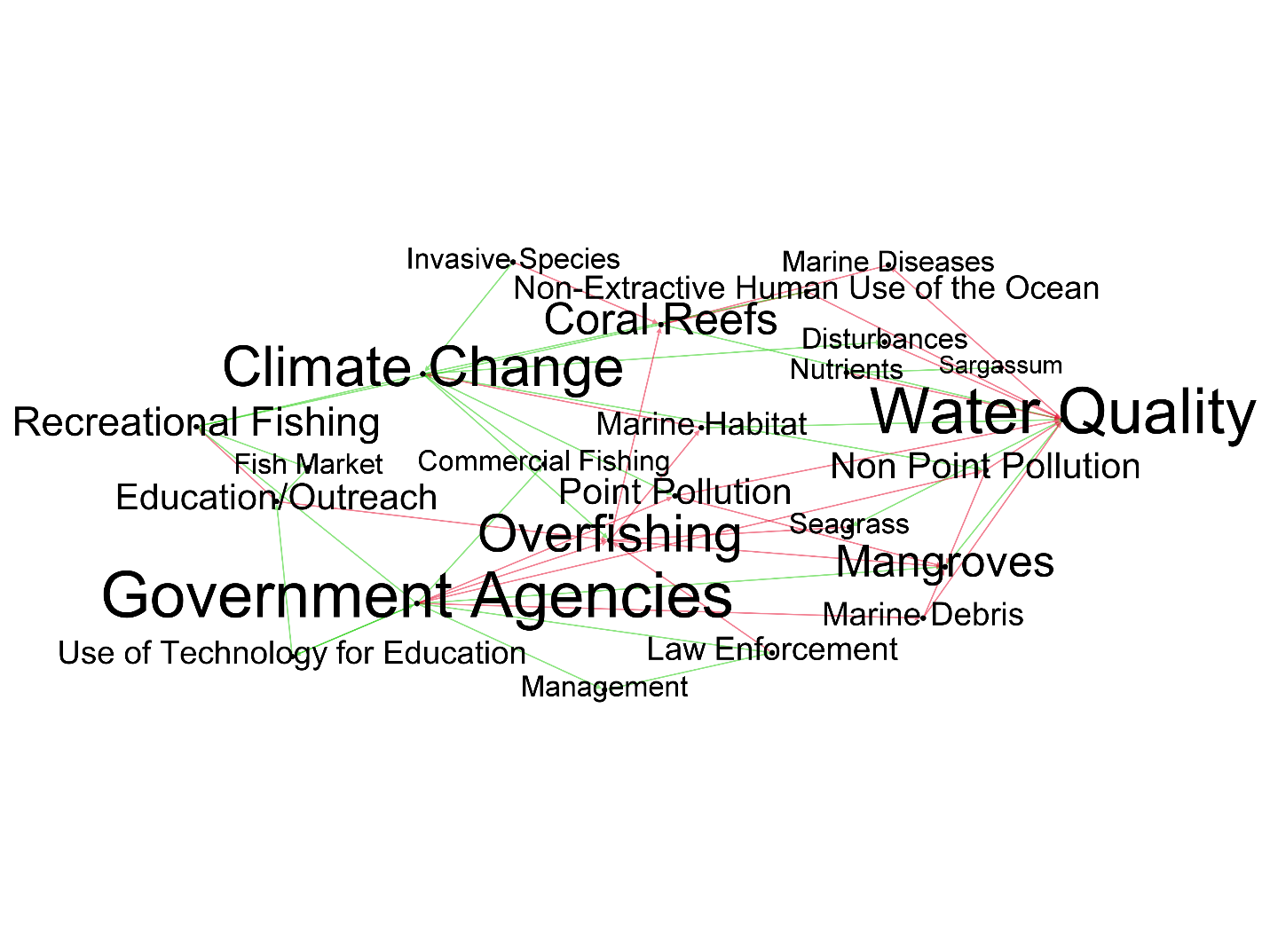


**S1 Fig 23. St. Croix NGOs Conceptual Model.**
