# Supplementary material for: Development and analyses of stakeholder driven conceptual models to support the implementation of ecosystem-based fisheries management in the U.S. Caribbean
Source: PLoS One. 2024 May 31;19(5):e0304101. doi: 10.1371/journal.pone.0304101 (PMC11142612; doi:10.1371/journal.pone.0304101)
Supplement: S2 File — (DOCX) [file pone.0304101.s003.docx]

# Supporting Information 2


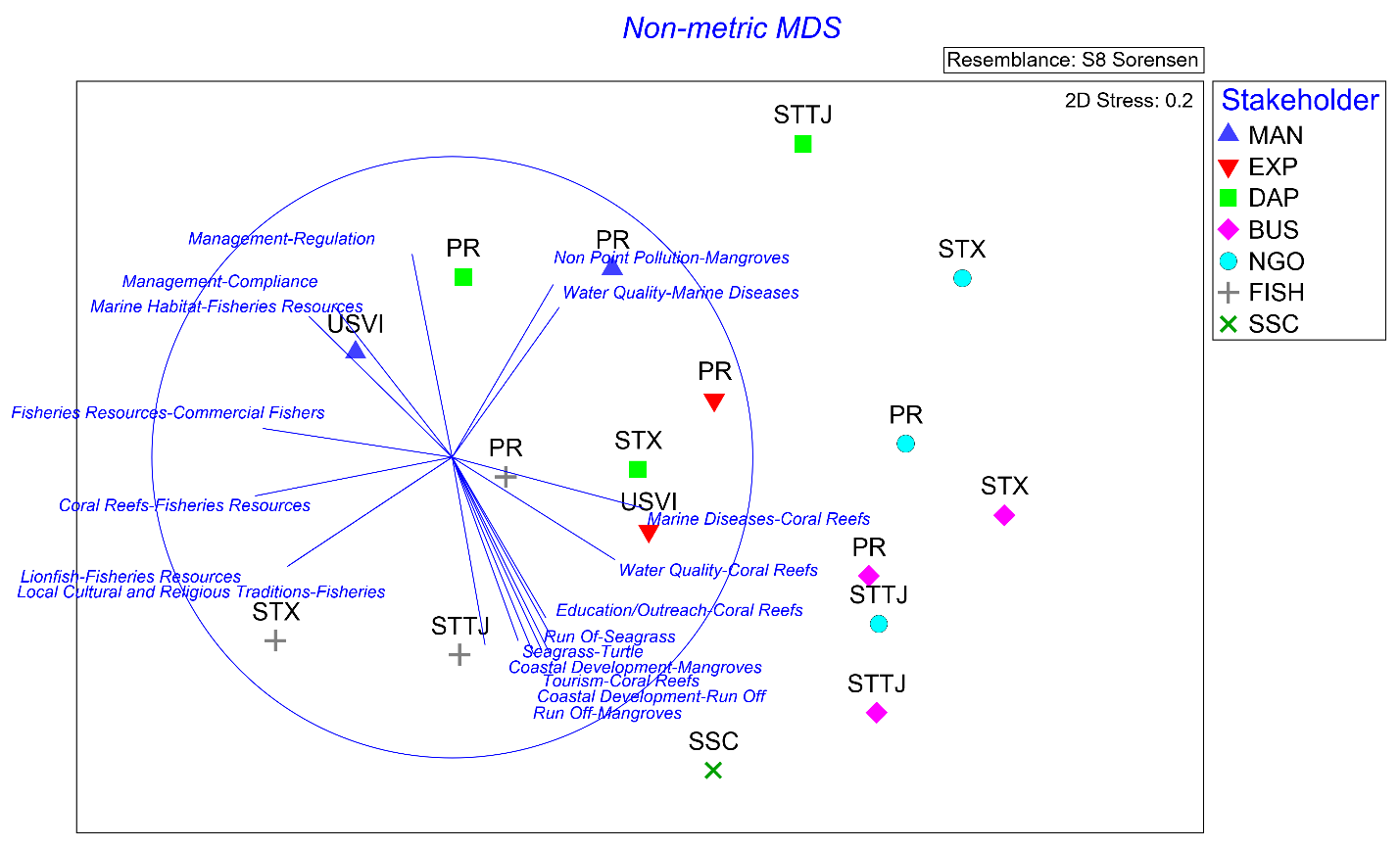


**S2 Fig 1 nMDS ordination of stakeholder groups and U.S. Caribbean islands based on a simple matching coefficient between relationships of ecosystem components identified in conceptual models. Most significant vectors appear in blue. (MAN=managers; EXP=academics; DAP=District Advisory Panels; BUS=businesses; NGO=Environmental Non-Governmental Organizations; FISH=commercial fishers; SSC=Scientific and Statistical Committee; PR = Puerto Rico; STTJ = St. Thomas/St. John, STX = St. Croix**
