# Supplementary material for: Development and analyses of stakeholder driven conceptual models to support the implementation of ecosystem-based fisheries management in the U.S. Caribbean
Source: PLoS One. 2024 May 31;19(5):e0304101. doi: 10.1371/journal.pone.0304101 (PMC11142612; doi:10.1371/journal.pone.0304101)
Supplement: S3 File — (DOCX) [file pone.0304101.s004.docx]

# Supporting Information 3


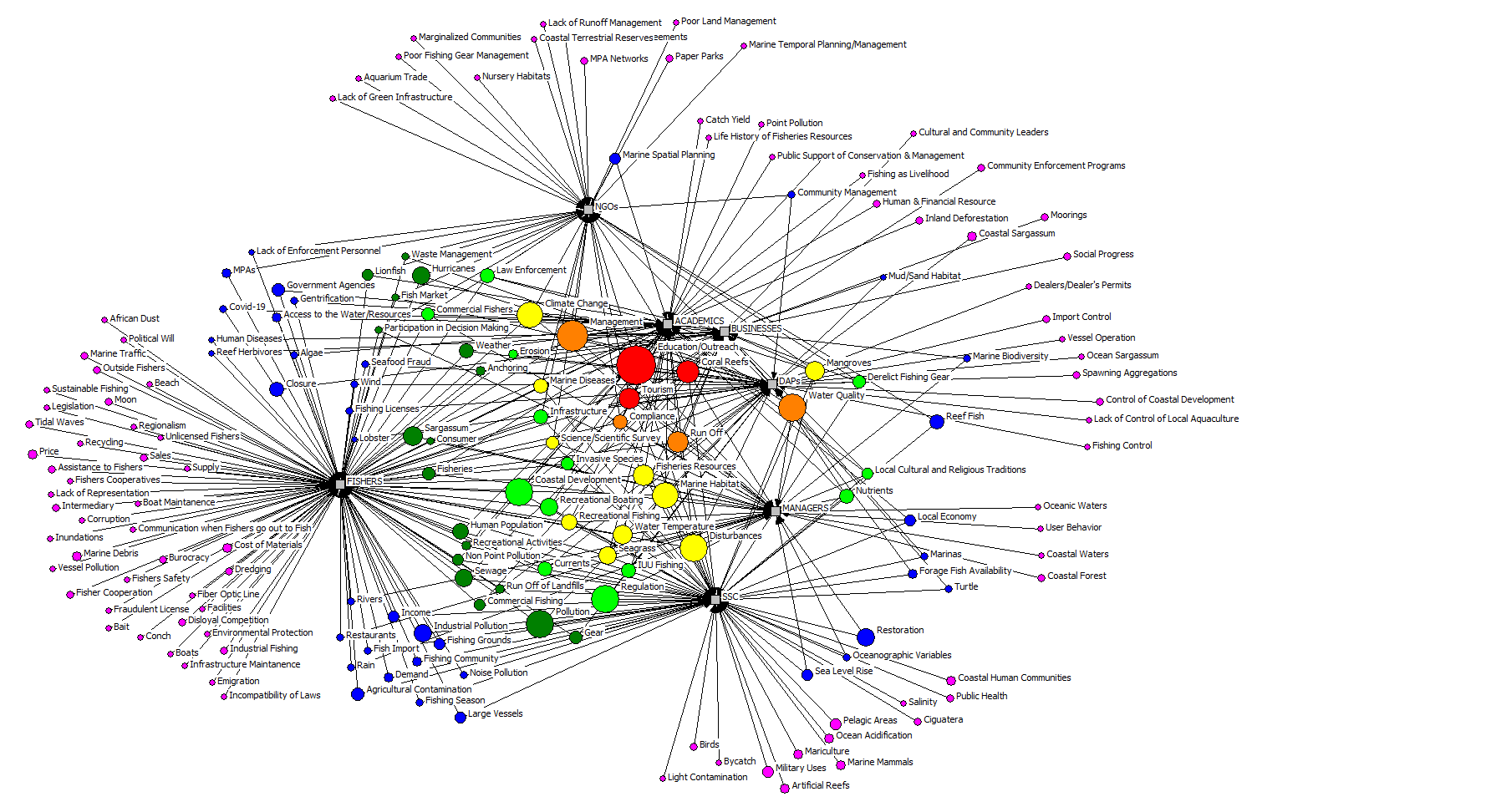


**S3 Fig 1. Puerto Rico Drivers 2-mode network analysis.**


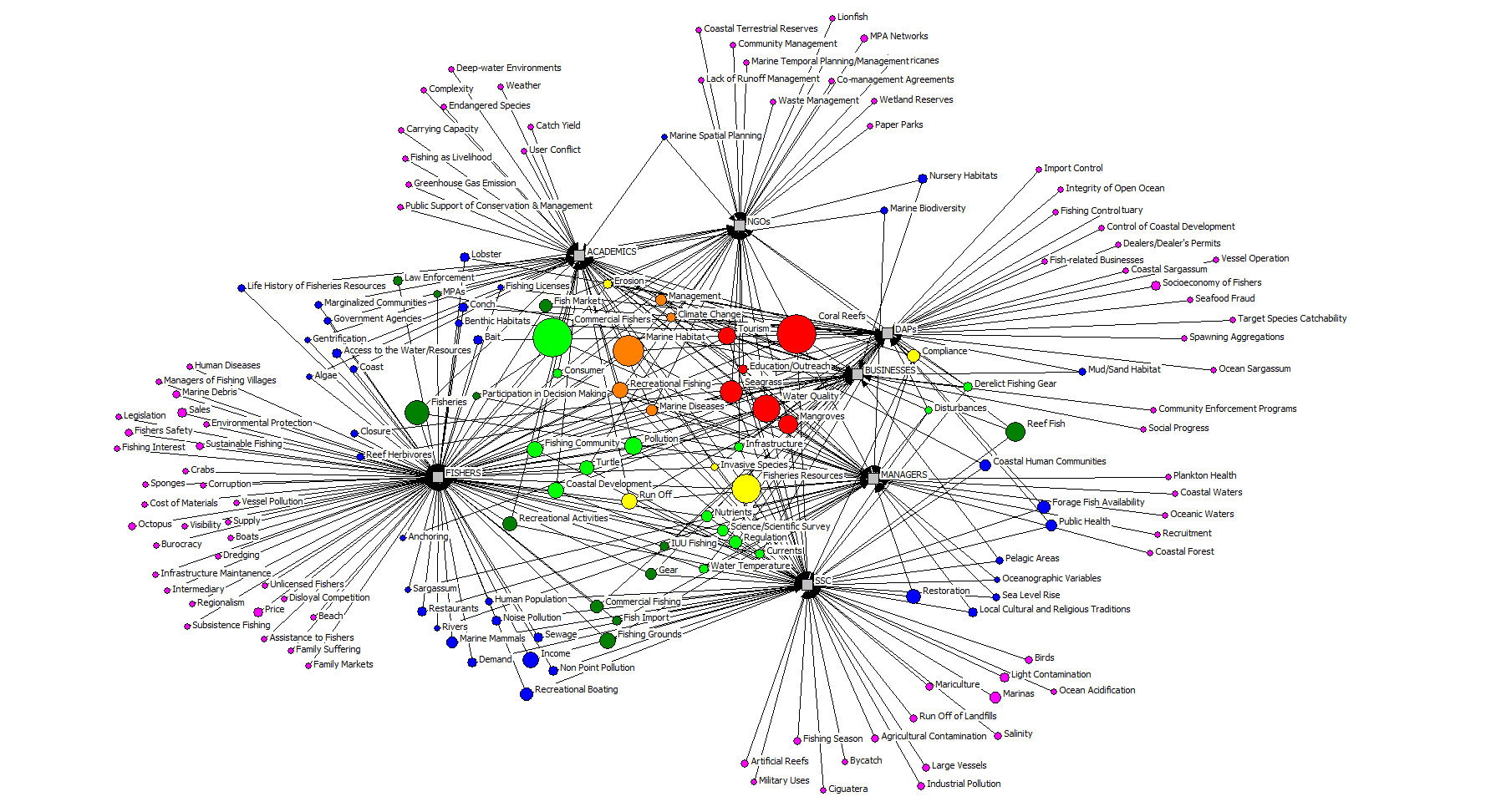


**S3 Fig 2. Puerto Rico Receivers 2-mode network analysis.**


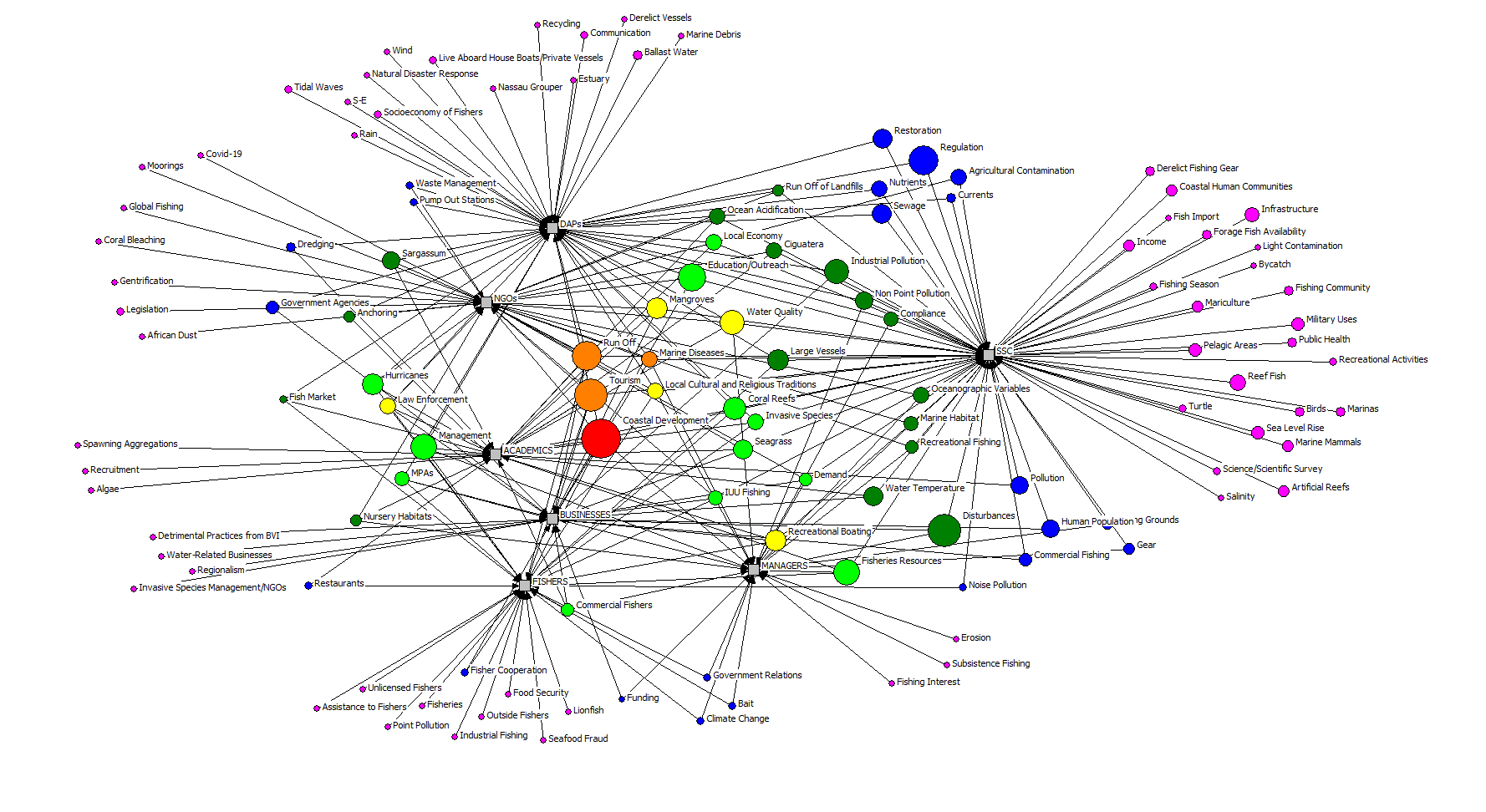


**S3 Fig 3. St. Thomas/St. John Drivers 2-mode network analysis.**


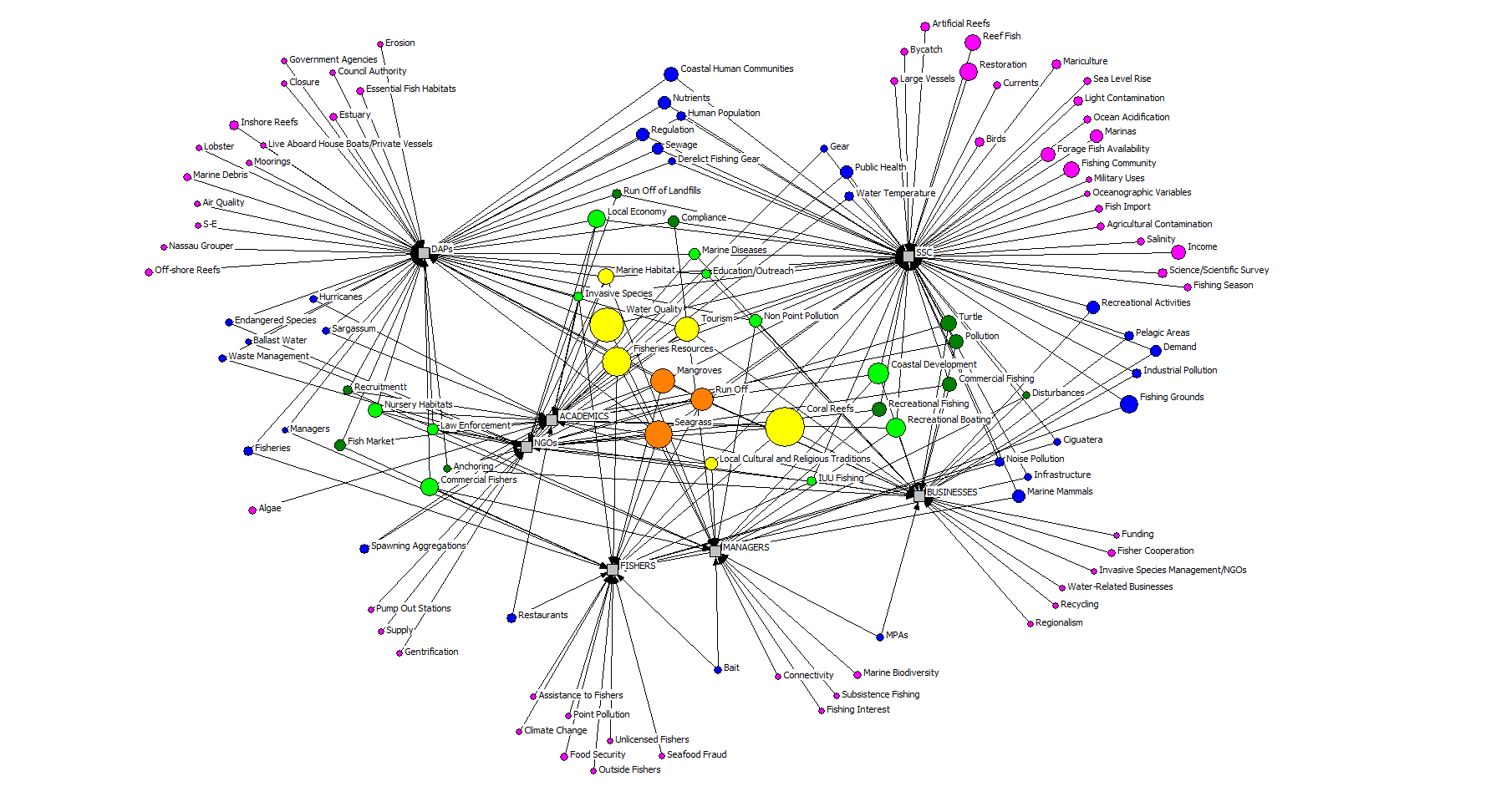


**S3 Fig 4. St. Thomas/St. John Receivers 2-mode network analysis.**


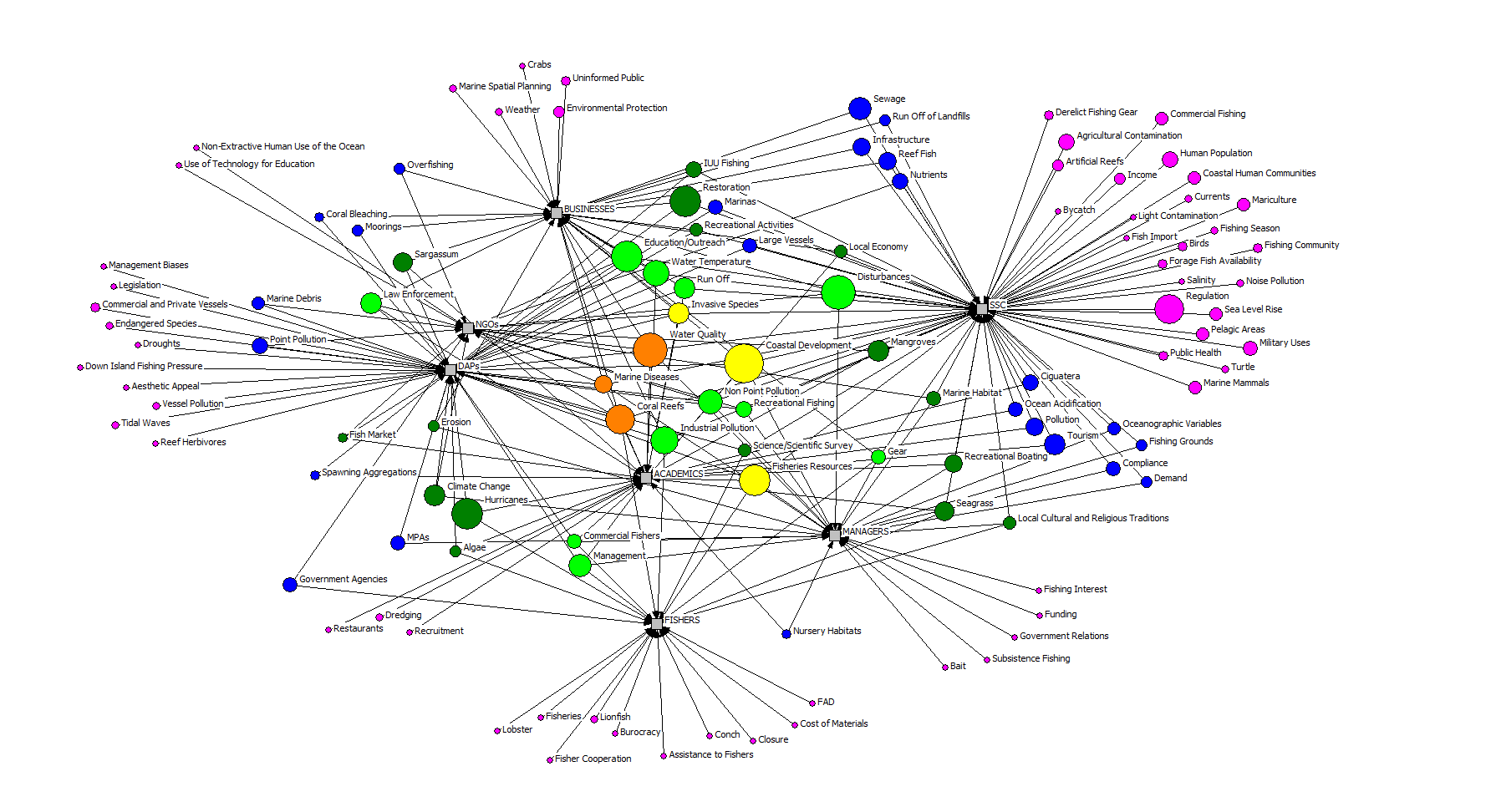


**S3 Fig 5. St. Croix Drivers 2-mode network analysis.**


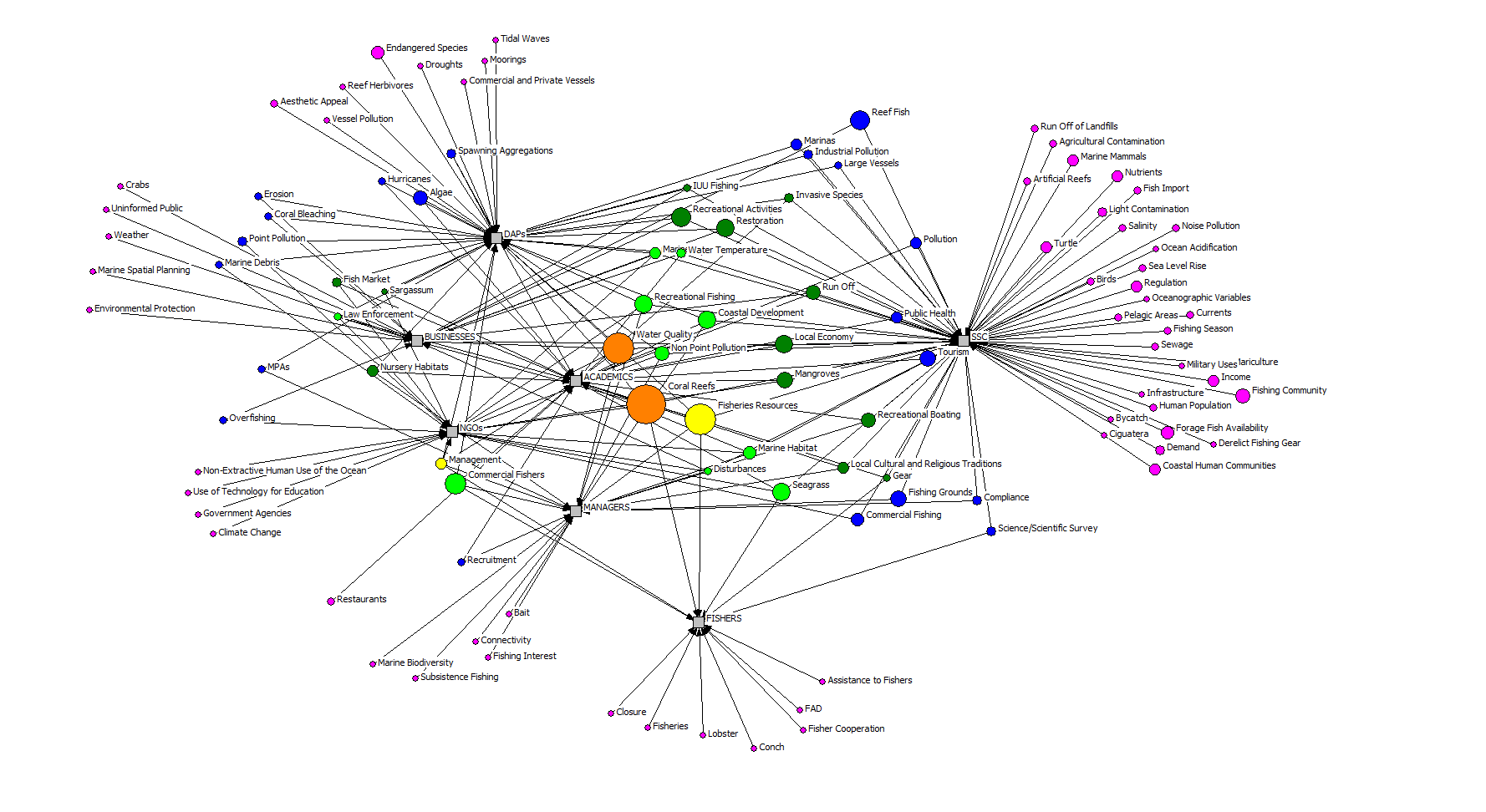


**S3 Fig 6. St. Croix Receivers 2-mode network analysis.**
